# Acknowledgment to Reviewers of *Nutrients* in 2020

**DOI:** 10.3390/nu13020382

**Published:** 2021-01-26

**Authors:** Nutrients Editorial Office

**Affiliations:** MDPI AG, St. Alban-Anlage 66, 4052 Basel, Switzerland

Peer review is the driving force of journal development, and reviewers are gatekeepers who ensure that *Nutrients* maintains its standards for the high quality of its published papers. Thanks to the cooperation of our reviewers, in 2020, the median time to first decision was 16 days and the median time to publication was 35 days. The editors would like to express their sincere gratitude to the following reviewers for their precious time and dedication, regardless of whether the papers were finally published:
Aagaard, Niels KristianLin, Xiaoxia (Nina)Aaron M., WalshLin, YanAarts, EstherLin, Yen-FengAas, Anne-MarieLin, Yi-TingAbadie, ValerieLin, Yuh-JyhAbalo, RaquelLin, Yung-ChiehAbate, GiuliaLinderborg, KaisaAbate, Maria LorenaLindley, MartinAbayomi, Julie C.Lindqvist, HelenAbbott, DavidLindsay, AngusAbdallah, AliLindsay, KarenAbdelmagid, SalmaLinert, WolfgangAbdel-Tawab, MonaLing, JiaxinAbenavoli, LudovicoLing, Pei-RaAbid, Muhammad BilalLio, DoAbitbol, Carolyn L.Liotti, AntoniettaAboul-Enein, BasilLiotto, NadiaAbraczinskas, MichelleLipiński, PawełAbraham, Elizheeba ChristieLipowska, MałgorzataAbrahams, MarietteLips, PaulAbrahamsson, Thomas R.Lipsky, Leah M.Abramyan, JohnLiras, Ioannis N.Abreu, SandraLiskiewicz, DanielaAbt, Michael C.Listrat, AnneAbushouk, AbdelrahmanLitchford, Mary D.Abuzaid, AhmedLiu, Bing-LanAchraf, AmmarLiu, BoAchterberg, Wilco P.Liu, BuyunAcosta, StefanLiu, ChangqiAcquah, CalebLiu, Chin-HungActon, RachelLiu, Chin-YuAdamczyk, GretaLiu, HaoyuAdamczyk, PiotrLiu, HongxiangAdamek, AgnieszkaLiu, JingAdams, Drew J.Liu, JueAdams, IngridLiu, Ken H.Adams, Katherine P.Liu, ManAdamska, AgnieszkaLiu, MengyangAdamska-Patruno, EdytaLiu, NanaAdan, AnaLiu, NingAddepalli, BalasubrahmanyamLiu, Richard T.Addison, CliftonLiu, YanAddo, YawLiu, Yu-HueiAdekanmbi, Victor T.Liu, YunAdekoya, Timothy O.Liu, YunfengAden, KonradLiu, ZhipengAdhikari, KoushikLiu, ZijuanAdhikari, RajanLivesey, GeoffreyAdibi, SasanLlanes, CarlosAdjé, FélixLlauradó, ElisabetAdom, TheodosiaLlopis, JuanAdriaens, Michiel E.Llopis-González, AgustínAe, RyusukeLlorente Cantarero, Francisco JesusAeddula, Narothama ReddyLloyd, PamelaAerenhouts, DirkLo Buglio, AurelioAffoo, RebeccaLo Giudice, AntoninoAffourtit, CharlesLo, Chunmin C.Afonso, AndreaLo, Shyh-ChyiAfrin, SadiaLobbes, HervéAgakidis, CharalamposLobo, DileepAgarwal, PujaLococo, Peter M.Aggarwal, Bharat B.Locquet, MédéaAggeli, Ioanna-KaterinaŁodyga-Chruścińska, ElżbietaAggett, Peter J.Loffredo, LorenzoAghajafari, FaribaLofgren, IngridAghajanyan, AnushLohner, SzimonettaAgho, Kingsley E.Lohse, BarbaraAgnello, MelissaLoiselle, Denis S.Agoro, RafiouLoizzo, JamieAgostini, DeborahŁojko, DorotaAgostinis, ChiaraLokesh, Belur R.Aguilar, AliciaLoman, BrettAguilar-Alvarez, DavidLoman, Brett R.Aguilar-Toala, Jose E.Lombardo, MauroAguilera, MargaritaLomonaco, TommasoAguirre, AitorLonardo, AmedeoAgyei, DominicLoncoman, CarlosAhamad, MazbahulLondon, EdraAhluwalia, PankajLong, Michael A.Ahmad, KhurshidLong, TaoAhmad, ShafqatLongo, MiriamAhmad, ShakilLongo, ValentinaAhmad, TanveerŁoniewska, BeataAhmed, MavraLönnerdal, BoAhmet, IsmayilLoo-Bouwman, Carolien Annika vanAhn, Eun KyungLoots, GabrielaAhn, Mok-RyeonLópez Aliaga, María InmaculadaAhn, Sang-BongLópez Malo, DanielAhola, AilaLopez Seijas, JacoboAhrens, Richard A.Lopez, David S.Ahrens-Nicklas, Rebecca C.López, Lucía MéndezAhuja, NitinLópez-Ejeda, NoemíAi, YongLópez-Galán, BelindaAisa, Maria CristinaLópez-Gil, José FranciscoAit-Ghezala, GhaniaLópez-Gómez, Juan JoséAkatsu, HiroyasuLopez-Miranda, JoseAkbulut, CengizLópez-Miranda, VisitaciónAkers, JeremyLópez-Mora, ClaraAkison, LisaLópez-Parra, Ana M.Akombi, Blessing J.Lopez-Samanes, AlvaroAkombi-Inyang, BlessingLopitz Otsoa, FernandoAlaedini, ArminŁopusiewicz, ŁukaszAlalwan, TariqLordan, RonanAlamiri, NasserLorente-Cebrián, SilviaAlañon, ElenaLorenzo, MicheleAlaofè, HalimatouLorenzo-Gómez, Maria-FernandaAlbaladejo-Blázquez, NataliaLorenzo-López, LauraAlbanes, DemetriusLoria Kohen, VivianaAlbaugh, VanceLossa, SusannaAlbracht-Schulte, KembraLossius, AndreasAlbrechtsen, Nicolai Jacob WewerLosurdo, GiuseppeAlcala, MartinLoughman, AmyAldaco, RubénLouis, SandrineAlemu, Mohammed HussenLouise, DeldicqueAlexi, NikiLove, DaveAlfarouk, Khalid OmerLovejoy, DavidAl-Fatimi, MohamedLow, David A.Alfieri, CarloLow, MitchellAlfieri, SergioLowe, AdrianAl-Ghadban, SaraLowe, MichaelAli, AmanatLowe, NicolaAli, Md YousofLowin, TorstenAli, Mohamed M.Lozano Blasco, RaquelAlicandro, GianfrancoLozano-Lorca, MacarenaAlizargar, JavadLu, ChangyuanAl-Jawaldeh, AyoubLu, Chi-HuaAllaway, DavidLu, Da-WenAllegaert, KarelLu, Kuo-ChengAllen, SimonLu, Louise WeiweiAllen, Stephen C.Lu, QingyiAllerton, TimothyLu, XiaohuaAlleva, RenataLu, XiaoxiaoAllman, BrittanyLubas, ArkadiuszAllman-Farinelli, MargaretLubecka-Pietruszewska, katarzynaAlloh, FolashadeLubout, Charlotte M.A.Alloza, IraideLuca, Arnaud DeAlmansa, JosuéLucan, SeanAlmeida Palha, JoanaLucantoni, FedericoAlmeida, Maria JuditeLucau-Danila, AncaAlmenara, CarlosLucey, AliceAl-Mrabeh, AhmadLudwig, Iziar A.Almståhl, AnnicaLudy, Mary-JonAl-Najim, WerdLueangsakulthai, JirapornAl-Nakkash, LaylaLuis Ubago, JoseAlonso-Moreno, CarlosLuisetto, RobertoAlonso-Torre, SaraLuk, Hui-YingAlquier, ThierryLuk, Ian Y.Alsharairi, NaserLukaski, HenryAl-Shargie, FaresŁukaszyk, EwelinaAltamura, ClaudiaLukkahatai, NadaAltemimi, AmmarŁukomska, AgnieszkaAltieri, BarbaraLuna Maldonado, AurelioAltirriba, JordiLuo, HanqiAltosaar, IllimarLuo, TingAlvarez Fernandez, María AntoniaLuo, XianÁlvarez-Mercado, Ana IsabelLuo, ZonghuaAlvarez-Sala-Walther, Luis A.Lupfer, ChristopherAlvarez-Suarez, JoseLupiáñez, José A.Alvero Cruz, Jose RamonLupoli, RobertaAlves, MarcoLupușoru, RalucaAlvisi, Maria FrancescaLuque, VeronicaAlwahsh, SalamahLurz, EberhardAlwerdt, JessieLustgarten, MichaelAmadora Pomar, CatalinaŁuszczki, EdytaAmal, HaithamLütjohann, DieterAmarelli, CristianoLux-Battistelli, ChristineAmarger, ValerieLuyer, MishaAmatori, StefanoLuyer, Misha D. P.Amelio, DanielaLy, Sun YungAmengual, JaumeLykkesfeldt, JensAmezcua-Prieto, CarmenLynch, HeidiAmigo-Benavent, MiryamLynch, KrystalAmir, Achiya Z.Lynn, AnthonyAmirabdollahian, FarzadLyons, GrahamAmmann, JeanineLytle, Leslie A.Ammar, AchrafM. Ney, DeniseAmores Arrocha, AntonioM’Koma, AmosyAmoresano, AngelaMa, DavidAmorim, FabianoMa, EnboAmrein, KarinMa, SihuiAmund, DanielMa, Zheng FeeiAn, RanMaalouf, NaimAn, WonsukMaares, MariaAn, YuMacáková, KateřinaAnand, SwatiMacalister, HeatherAnania, Maria ChiaraMacchia, Paolo Emidio MidioAnderegg, UlfMacDonald-Wicks, LesleyAnders, John Paul V.MacDonell, SueAndersen, Barbara VadMace, ThomasAndersen, Jens RikardtMachado, YoanAndo, MasashiMachann, JürgenAndo, TakafumiMachida, DaisukeAndolina, Jeffrey R.Machin, Daniel R.Andrade, Eric FrancelinoMachoy, Monika ElżbietaAndrade, Fabia de OliveiraMacías-González, ManuelAndrade, Jeanette M.MacIejewska, DominikaAndrade, José CarlosMacín, Stella MarisAndrade, JuanMack, IsabelleAndreeva, Valentina A.Mackay, SallyAndreotti, GabriellaMackenbach, Joreintje DingenaAndrés, AnaMackenzie, Richard W.A.Andreucci, Maria BeatriceMackie, AlanAnestopoulos, IoannisMacMillan, FreyaAngele, ClaudiaMacnaughtan, JaneAngeli, TimMacNell, LillianAngelico, RobertaMadalina, IugaAngelino, DonatoMadej, DawidAngeloni, CristinaMadej, Jan PawelAngelopoulou, Matina V.Mademtzoglou, DespoinaAngileri, Flavio FilippoMader, SebastianAnguah, Katherene O.-B.Madireddy, AdvaithaAnifandis, GeorgeMaeda, HayatoAnjos, OféliaMaeda, KeisukeAnn Smith, HazelMaehre, HanneAnna Poulia, KalliopiMaestro, MiguelAnnarapu, Gowtham K.Magan-Fernandez, AntonioAnnibale, BrunoMaggio, LucaAnnunziata, GiuseppeMagierowski, MarcinAnsari, RaisMagini, AlessandroAntiga, EmilianoMagnano San Lio, RobertaAntoine-Jonville, SophieMagni, PaoloAntolak, HubertMagnone, MirkoAnton, PaulineMagriplis, EmmanuellaAntonella, SantonicolaMagrone, TheaAntoniewska, AgataMaguire, DonoghAntonoglou, Georgios N.Mahalingaiah, ShruthiAntonysunil, AdaikalaMahar, RohitAntosiewicz, Anti-oxidationJędrzejMaher, JudeAntunes, HenedinaMähler, AnjaAnyżewska, AnnaMahli, AbdoAoe, SeiichiroMahmud, IqbalAoki, MasahikoMai, VolkerAoki-Nonaka, YukariMaida, Carlo DomenicoApolzan, John W.Mailhot, GenevieveApone, FabioMair, JacquelineApostolovic, DanijelaMaisto, MariaApplegate, CatherineMaitz, ManfredAppleton, KatherineMajewski, MichałAppollonio, IldebrandoMajumder, AvisekAprile, Maria CarmelaMajumder, KaustavAprotosoaie, Ana ClaraMak, IvanAquino, Rita De CássiaMakarewicz-Wujec, MagdalenaAragón Vela, JerónimoMaki, KevinAragonès, GerardMakki, KassemArai, ToshiroMakris, Konstantinos C.Aran, GemmaMaksimov, SergeyAranceta-Bartrina, JavierMalaguarnera, LuciaArany, EdithMalaguarnera, MarianoArata, NaokoMalbert, Charles-HenriAraújo, JoanaMalczewska-Lenczowska, JadwigaAraujo, PedroMalfa, Giuseppe AntonioAraujo, RicardoMałgorzata, KowalskaArbillaga Etxarri, AneMalhotra, KunalArcher, DavidMalicka, IwonaArcher, EdwardMalik, PreetiArciero, PaulMalik, Rayaz A.Arcone, RosariaMalin, StevenArczewska, KatarzynaMalisova, OlgaArduini, ArduinoMallan, KimberleyArends, JannMallineni, Sreekanth KumarArevalo, EsterMaloberti, AlessandroArguelles, SandroMałodobra-Mazur, MałgorzataArgueta, DonovanManai, FedericoArgyrakopoulou, GeorgiaManary, MarkArgyropoulos, KonstantinosMañas, AsierAridi, Yasmine S.Mancuso, ElettraArif, TasleemMandal, AmritlalArikawa, Andrea Y.Mandala, AshokAriño, OlgaMandard, StéphaneAriyani, WindaMandato, ClaudiaAriza, Maria TeresaMandelbaum, DavidArjamaa, OlliMandolesi, SerenaArjan, NarbadMandreoli, MarcoraArmand, MartineManfredini, RobertoArmanini, DecioMangano, KatiaArmeni, EleniMangano, KelseyArmeni, TatianaMangge, HaraldArmentano, Maria FrancescaMangoni, Arduino A.Armitage, AndrewManiam, JayanthiArmstrong, HeatherManjunath, ManuboluArnaoutis, GiannisMankowska-Wierzbicka, DorotaArnold, JasonMankowski, Robert T.Aronsson, Carin AndrénMann, FrancisArosio, PaoloMannan, HaiderArroo, RandolphMannetje, AndreatArtero, SylvaineMannucci, CarmenArtunc, FerruhManoogian, EmilyAruanno, AriannaManor, DannyArunkumar, AbhiramManore, MelindaAsadipooya, KamyarMansell, TobyAsano, ShinichiMansoorian, BaharehAscani, AngeloMansour, SherryAseervatham, JayaManthou, EiriniAsher, JordiMantilla Herrera, Ana MariaAshorn, UllaMantzioris, EvangelineAsim, MuhammadManzo, CiroAssadi-Porter, Fariba M.Mapelli, MassimoAssanelli, DeodatoMarabini, LauraAssirelli, ElisaMarakis, GeorgiosAstasio-Picado, ÁlvaroMaranghi, FrancescaAstbury, StuartMarasco, GiovanniAsztalos, ElizabethMarathe, ChinmayAtawia, Reem T.Marathi, RachanaAthanasiadis, VassilisMarazziti, DonatellaAthanassakis, IreneMarciniak-Łukasiak, KatarzynaAthinarayanan, ShaminieMarconi, Anna MariaAthrey, GiridharMarcos, PilarAtteritano, MarcoMarculescu, RodrigAttuquayefio, TukiMardas, MarcinAuerbach, MichaelMarek, KieliszekAuguet, TeresaMargaglione, MaurizioAugustin, HannaMargalef, MariaAugustine, JosyMargalit, IliAunsholt, LiseMargaritelis, NikosAuricchio, SalvatoreMargaritis, IrèneAvci, RecepMargerison, ClaireAversa, AntonioMargolis, LeeAvgerinou, ChristinaMari, DanielaAvila-Román, JavierMaria Donini, LorenzoAvin, VijayMaria Irene, BelliniAvonto, CristinaMaría Rosaura, Leis TrabazoAvtanski, DimiterMarian, BrigitteAxelrod, Cara HannahMarin, DiegoAxiotis, EvangelosMarinangeli, Christopher P.F.Ayad, AmiraMarín-García, Pablo JesúsAydede, Sema K.Marini, Herbert RyanAydemir, TolunayMarini, Juan C.Ayers, PhilMarino, JosephAyonrinde, OyekoyaMarkaki, AnastasiaAyo-yusuf, Olalekan A.Markowiak, PaulinaAyton, AgnesMarkowicz, SergiuszAyuso, Juan MielgoMarks, Emma J.Azam, Aa HaerumanMarletta, LuisaAzarnia Tehran, DomenicoMármol, InesAziz, AtharMarotta, ClaudiaAziz, ImranMarotte, HubertAzzimonti, BarbaraMarousez, LucieAzzolini, ClaudioMarques, CláudiaAzzolino, DomenicoMarques-Lopes, IvaBaack, MichelleMarrano, NicolaBabickova, JankaMarreiros, CatarinaBabio, NancyMarriott, Bernadette P.Babu, DineshMarrodán Serrano, María DoloresBacchetti, TizianaMarrodan, DoloresBachou, HanifaMarron, MeganBadeau, Robert M.Marrone, GiuseppeBae, Hae-RahnMarshall, AutumnBae, WonMarsili, StefaniaBae, YangjinMarsol, AlexisBaerga-Ortiz, AbelMartellucci, StefanoBaffour, Kwaku KyeiMartens, ChristopherBagchi, DebasisMartín Bermudo, Francisco ManuelBagüés, AnaMartín Hernández, RobertoBagyinszky, EvaMartin, DanBahorski, JessicaMartin, KeithBai, LiMartin, NeilBai, RenrenMartin, PatrickBaile Ayensa, José IgnacioMartín, RafaelBailey, CateMartin, Robert C.G.Bailey, HelenMartin, StephanBailey-Davis, LisaMartin, Stephanie L.Baird, Danielle LouiseMartin-Calvo, NereaBais, BabetteMartínez Beamonte, RobertoBaïz, NourMartinez Calejman, CamilaBajaj, PuneetMartínez Graciá, CarmenBajka, BalazsMartinez, HomeroBaker, Brent A.Martinez, MercedesBakht, MartinMartínez, Silvia VarelaBakillah, AhmedMartínez-Cañamero, MagdalenaBakker, Mieke H.Martínez-Espinosa, Rosa MaríaBakoyiannis, IoannisMartínez-Galiano, Juan MiguelBalakrishnan, BijuMartínez-García, AlbaBalawejder, MaciejMartínez-García, M. ÁngelesBalcerczyk, AnetaMartinez-Guryn, KristinaBaldassano, SaraMartinez-Hervas, SergioBaldassarre, Maria ElisabettaMartínez-Nova, AlfonsoBalk, EthanMartínez-Ortega, Antonio JesúsBalland, EglantineMartínez-Rodriguez, AlejandroBallco, PetjonMartínez-Sánchez, GregorioBallestri, StefanoMartínez-Sánchez, NoeliaBallini, AndreaMartinez-Tellez, BorjBaloni, PriyankaMartini, DanielaBalsano, ClaraMartín-Montalvo, AlejandroBalsinde, JesusMartino, GabriellaBalzano, TizianoMartino, Giuseppe DiBamba, Bio Sigui BrunoMartinotti, SimonaBamba, ShigekiMartins, ArturBambling, MatthewMartins, Maria JoãoBammann, KarinMartins, NatáliaBancirova, MartinaMartins, PauloBandyopadhyay, BidhanMartín-Sánchez, Francisco JavierBanerjee, SudipMartins-Oliveira, MargaridaBanik, AnnaMartone, Anna MariaBanks, Alexander S.Marty, LucileBannenberg, GerardMartyn, DanikaBansode, Rishipal R.Martynowicz, HelenaBao, YongpingMarugán-de-Miguelsanz, Jose ManuelBapputty, ReenaMarunaka, YoshinoriBaptiste-Roberts, KeshaMaruvada, PadmaBarad, DavidMaruyama, TakayukiBarak, MiraMarx, Joannes J. M.Baran, JoannaMary, M. MurphyBaranov, Maksim V.Marzec, AgataBaranowski, ThomasMarzullo, PaoloBarbalace, Maria CristinaMasaki, TakayukiBarbalho, Sandra MariaMascie-Taylor, NicholasBarbarossa, Iole TomassiniMaseda, Ana B.Barbeau, WilliamMasento, NatalieBarber, ElizabethMaslin, KateBarberio, BrigidaMason, RebeccaBarbosa, MarianaMason, Tyler BBarbosa-Yañez, Renate LuzíaMassad, Salwa G.Bárcena, CleaMassadi, Omar AlBarchetta, IlariaMassaro, MarikaBarclay, Sarah F.Massart, AlainBarda, NoamMassimini, MarcellaBardag-Gorce, FawziaMastana, SarabjitBardají, EduardMasterson, Travis D.Bardova, KristinaMastroianni, AntonioBärebring, LinneaMastroleo, FeliceBareja, AkshayMasuzaki, HiroakiBarffour, MaxwellMatacchione, GiuliaBarja, SalesaMatafome, PauloBarker, TylerMate, AlfonsoBarley, OliverMatencio, AdriánBaron, MorganeMateos Bernal, Rosa MariaBarone, Maria VittoriaMateu De Antonio, JavierBarone, MicheleMateus, SóniaBaroni, LucianaMathai, PrinceBaroni, StefanoMatheson, BrittanyBarrachina, Maria DoloresMatheson, JesseBarrada, Juan RamónMathew, Roy O.Barragán, RocioMathieu, JulieBarratt, MichaelMathis, Mary WBarrea, LuigiMathisen, Therese FostervoldBarreiro-Hurlé, JesúsMatiadis, DimitriosBarreto, GoncaloMatias, CatarinaBarrett, Robert J.Matinolli, Hanna-MariaBarrios-Rodríguez, RocioMatłosz, PiotrBarros, AnaMatsubara, KeiichiBarros, Joana M.Matsui, TakashiBarros-Dios, Juan MiguelMatsumoto, TakayukiBarshtein, GregoryMatsumoto, YasuhikoBarszcz, MarcinMatsumoto, YoshinoriBartelt, AlexanderMatsumura, TsuyoshiBarthels, FriederikeMatsushita, TakehikoBartolomei, SandroMatsuura, ShinobuBartolomeo, GiovanniMatsuzaki, KentaroBarton, KarenMatsuzaki, MikaBarton, Larry L.Matsuzaki, ShinyaBartosz, GrzegorzMatta, JoaneBartuzi, ZbigniewMattei, JosiemerBarzilay, JoshuaMatthews, Joseph J.Basak, SanjayMatthys, ChristopheBasak, Saroj K.Mattii, LetiziaBasak, SujitMattioli, Anna VittoriaBasic, MarijanaMattner, JochenBasili, StefaniaMatusiewicz, MalgorzataBasini, GiuseppinaMaugeri, AlessandroBasiri, RaedehMaugeri, AndreaBaskaran, RathinasamyMaugeri, Andrea GiuseppeBasnet, SijanMaurer, StefanieBassi, Anna MariaMauricio, Maria DoloresBastone, Alessandra De CarvalhoMauriello, GianluigiBasu, ArpitaMaurya, ShailendraBaswan, SudhirMavropoulos, AthanasiosBatacan, RomeoMawson, AnthonyBatal, MalekMay, JamesBathrellou, EiriniMayengbam, ShyamchandBatista, EraldoMayeuf-Louchart, AliciaBatra, NeeluMayneris-Perxachs, JordiBattafarano, GiuliaMayr, Johannes A.Battah, BasemMayra, SeliciaBaum, ThomasMays, Jacqueline W.Baumann, AnjaMays, RyanBaumgard, LanceMazanowska, NataliaBaumgartner, SabineMazidi, MohsenBavaro, SimonaMazumder, KishorBayer, SimoneMazur, ArthurBaylin, AnaMazur-Bialy, AgnieszkaBazacliu, CatalinaMazurek, DominikaBazylko, AgnieszkaMazzocchi, AlessandraBeal, TyMcAllister, MattBeals, Daniel A.McArdle, SaraBeard, MatthewMcArthur, SimonBeasley, SheaMcBurney, MichaelBeauchamp, GaryMcCaffery, PeterBeaudart, CharlotteMcCann, AdrianBecerra-Tomás, NereaMcCarthy, ElaineBecker, GenevieveMcCarthy, HelenBecker, SebastianMcCarthy, Sinéad N.Beckett, EmmaMcCartney, DanielBecquey, ElodieMcCarty, CatherineBedard, AnnabelleMcClain, AmandaBegdache, LinaMcCloat, AmandaBeggiato, SarahMcClure, ScottBegley, AndreaMcCluskey, LynnetteBehrens, MartinMcComb, JacalynBeier, JulianeMcConell, GlennBein, AmirMcCourt, PeterBekaert, BramMcCullough, FionaBélanger, MélissaMcCully, KilmerBelén Ruiz-Roso, MaríaMcCurdy, KarenBelenguer, AlvaroMcDaniel, Marisol D.Bell, ColinMcDermott, LindsayBell, Jack J.McElrone, MarissaBell, Katherine A.McElroy, Steven J.Bell, WinnieMcFadden, JosephBellar, DavidMcFarland, Lynne V.Bellavia, DanieleMcGale, Lauren SophieBellelli, RobertoMcGee-Lawrence, MeghanBellido Guerrero, DiegoMcGill, Mitchell R.Bellini, MassimoMcGinnis, Graham R.Bellisle, FranceMcGrath, Kathleen H.Bello, Nicholas T.McGrath, KristineBello-Espinosa, Luis E.McGregor, Neil R.Belluzzi, ElisaMcGuire, MichelleBel-Serrat, SilviaMcGuire, ShelleyBelski, ReginaMcHill, Andrew WBelval, LukeMcilroy, GeorgeBenatar, Jocelyne R.McInnes, NataliaBendavid, ItaiMcIntosh, WilliamBendich, AdrianneMcIntyre, Jeremy C.Bendik, IgorMcJarrow, PaulBendix, MiaMcKay, Naomi J.Benedict, ChristianMcKendry, JamesBenfaremo, DevisMcKenna, Malachi J.Benítez-Cabello, AntonioMcKenna, MaryBenjamin, CourteneyMcKenna, Peter EdwardBennardo, LuigiMcKenzie, BriarBennasar Veny, MiguelMcKinley, MichaelBennetau-Pelissero, CatherineMcKinney, Matthew StuartBennett, Annemarie E.McLachlan, StelaBennett, ChristieMcLean, RachaelBenninghoff, AbbyMcLeod, GeriBen-Tovim, DavidMcLeod, JonathanBenzeroual, Kenza E.McMahan, ZsuzsannaBeppu, FumiakiMcMahon, GerardBerardi, EmanueleMcManus, AlexandraBerardi, RossanaMcManus, KirkBerardo, ClarissaMcMartin, KennethBerardy, AndrewMcNulty, JenniferBerding, KirstenMcRae, Marc P.Bereza-Malcolm, LaraMcSweeney, Matthew B.Berg, Akiah OMd Badrul, AlamBergamaschi, GaetanoMeans, Robert T.Berg-Beckhoff, GabrieleMearns, GaelBerger, MetteMeccariello, LuigiBerges, Gabriel LozanoMedawar, EvelynBergner, ErynnMedici, ValentinaBernabe, PenalverMędrzycka, WiolettaBernabe-Ortiz, AntonioMeek, Claire L.Bernard, JonathanMeerza, Syed Imran AliBernardini, ChiaraMeesters, DennisBernhard, WolfgangMehariya, SanjeetBerni Canani, RobertoMehta, Gaurav A.Bernstein, MelissaMehta, JawaharBerral, FranciscoMei, Hui-ChingBerrington, JanetMeir, MichaelBerry, Ann AndersonMeiselman, Herbert L.Berryman, ClaireMeisinger, ChristaBertani, LorenzoMelaku, YohannesBertea, CinziaMelanson, KathleenBerteau, Jean-PhilippeMelgar, SilviaBerthold-Pluta, AnnaMelini, FrancescaBerthoz, SylvieMelis, MelaniaBerti, AlviseMelkani, Girish C.Bertini, MarcoMelloul, EmmanuelBertolo, RobertMelnik, BodoBertone-Johnson, Elizabeth R.Melo, Bernardete F.Besser, GeroldMeltzer, Helle MargreteBest, RussellMena, SalvadorBetemariam, Senait AshenafiMenal, SusanaBettaieb, AhmedMenanteau-Ledouble, SimonBettendorff, LucienMena-Tudela, DesiréeBettinelli, Maria EnricaMendes, Roberta HackBeversdorf, DavidMendes, RosaBeydoun, May A.Méndez-Álvarez, EstefaníaBezirtzoglou, EugeniaMéndez-Martínez, CarlosBhagirath, DivyaMendonça, NunoBhandari, Manohar PrasadMeneguzzo, FrancescoBhandarkar, NikhilMeneguzzo, PaoloBhanji, Rahima A.Menendez, JavierBharaj, PreetiMeng, HongdaoBharath, Leena P.Meng, WeihuaBhardwaj, GouravMeng, YingBhat, JaydeepMeng, YuBhat, KruttikaMenghini, LuigiBhat, ShridharMennella, IlarioBhatia, DivyaMenni, ChristinaBhattacharya, AnannyaMenon, KavithaBhurosy, TrishneeMenon, PurnimaBiałecka-Dębek, AgataMensah, Enoch A.Białek, AgnieszkaMercadal, LucileBiałek, MałgorzataMercader, JosepBiałopiotrowicz, EmiliaMerecz-Sadowska, AnnaBianchi, Laurie M.Merendino, NicolòBickerdike, AndreaMergia, AyalewBidoli, EttoreMerino-Andres, JavierBidzan, MariolaMerkiel-Pawłowska, SylwiaBiedermann, DavidMerkler, DavidBiehl, Stefanie C.Merli, ManuelaBielczyk-Maczyńska, EwaMerlino, Valentina MariaBienertová-Vaškü, Julie AnnaMerlo, Lisa J.Biesiada, AnitaMero, AnttiBigiani, AlbertinoMerritt, RowenaBihuniak, Jessica DauzMerritt, Russell J.Bijak, MichałMerz, BenediktBikbova, GuzelMessaraa, CyrilBilancio, AntonioMessenio, DarioBilleter, AdrianMessina, MarkBillingsley, Hayley E.Messini, Christina I.Bilski, JanMetcalfe, RichardBimbo, FrancescoMethenitis, SpyridonBin, Yu SunMetyas, SamyBINDELS, LaureMetz, LoreBinder, AliceMetzger, Herr PhilippBird, Julia K.Meucci, MarcoBird, StephenMey, JacobBirgegård, AndreasMeyer, Alexa LeonieBisbal, CatherineMeyer, BarbaraBiscetti, FedericoMeyer, Haakon E.Bisch, StevenMeyer, SharonBishwajit, GhoseMeyer, SonyaBisti, SilviaMeyer, TimBistrian, BruceMeynier, AnneBito, TomohiroMian, CaterinaBitto, AlessandroMiao, JiBiver, EmmanuelMicarelli, AlessandroBizzaro, DeboraMicek, AgnieszkaBjerregaard, Anne AhrendtMiceli, Mathieu DiBjoernsbo, Kirsten SchrollMichaelidou, Alexandra-MariaBjørke Monsen, Anne-LiseMichaelsen, Kim F.Björkhem-Bergman, LindaMichaëlsson, KarlBkaily, GhassanMichalak-Majewska, MonikaBlack, Dennis D.Michel, Martin ChristianBlackmore, IvyMichel, PiotrBlades, MabelMichels, AlexBlais, AnneMichels, AlexanderBlanco Blanco, JoanMichels, Alexander J.Blanco-Gandía, M.CarmenMichishita, RyomaBlaney, SoniaMichos, Athanasios GBlanton, CynthiaMicioni Di Bonaventura, Maria VittoriaBlasetti, AnnalisaMickael, Michel-EdwarBlasiak, JanuszMickiewicz, AgnieszkaBłaszczyk-Bębenek, EwaMiddleton, Philippa F.Blat, SophieMiele, MaraBlechert, JensMielgo-Ayuso, JuanBleizgys, AndriusMigdalis, IliasBlekkenhorst, LaurenMiggiano, Giacinto Abele DolnatoBlesson, Chellakkan SelvanesanMigliaccio, SilviaBlicharska, ElizaMiguez, PatriciaBlicharski, TomaszMika, AdrianaBloch, PaulMikhailov, Theresa A.Blom, BiancaMikołaj, KamińskiBloomfield, FrankMikropoulos, ChristosBlumenfeld Olivares, Javier AndresMikšić, ŠteficaBo, SimonaMilagro, FerminBoakes, Robert A.Milan, AmberBoard, MaryMilani, ChristianBoaz, MonaMilani, Gregorio PaoloBobrowska-Korczak, BarbaraMilatz, SusanneBobulescu, Ion AlexandruMilczarek, MagdalenaBocarsly, MiriamMilenkovic, DraganBoccardi, VirginiaMiles, AnnaBodin, JohannaMiletta, MariaBodnar, WandaMilhano Santos, FátimaBodnar, ZsoltMilic, NatasaBody-Malapel, MathildeMillar, CourtneyBogard, JessicaMillar, John S.Bogdański, PawełMillar, LynneBogl, Leonie HelenMillar, SeánBöhm, VolkerMillen, AmyBohn, TorstenMiller Jones, JulieBoland, SebastianMiller, Gary D.Bolasco, PiergiorgioMiller, JacquelineBold, JustineMiller, LarryBollag, WendyMiller, LaurieBollong, MichaelMills, CharlotteBolton, Jessica L.Mills, ChristineBolton, KristyMills, JamesBoluda Ramos, EsterMills, Jennifer S.Bombardi, CristianoMilne, Barry JohnBömer, NilsMilner, Erin M.Bonaccio, MarialauraMilton, IñakiBonacina, FabriziaMin, HyeyoungBonadonna, AlessandroMin, JungwonBonam, Srinivasa ReddyMin, KisukBonato, MatteoMin, ThinzarBonaventura, AldoMin, Won LeeBonavina, LuigiMin, Yang WonBone, JuliaMinard, Charles G.Bonfrate, LeonildeMinaya, DulceBongers, KaleMiniati, MarioBonilla, CarolinaMiniero, RobertoBonior, JoannaMinihane, Anne MarieBonofiglio, DanielaMinistrini, StefanoBonora, MassimoMiotto, PaulaBoo, Yong ChoolMir, SartajBoolani, AliMiralles, BeatrizBoone, SebastiaanMiranda, JonatanBorczak, BarbaraMiranda, Jose M.Bordejé Laguna, Maria LuisaMiranda-Ackerman, Marco A.Bording-Jorgensen, MichaelMire, ErikBordonaro, MichaelMirtallo, Jay M.Bordoni, LauraMirzaei, SiroosBorengasser, Sarah J.Mirzakhani, HoomanBorer, Katarina T.Misawa, ErikoBorg, Stephanie A.Mishra, Jay S.Borghini, RaffaeleMishra, ManishBorja, Judith B.Mishra, MeerambikaBorkowska, Edyta MartaMishra, MuditBortkiewicz, AlicjaMishra, Santosh K.Bos, GerardMissel, AmandaBoschmann, MiachaelMistlberger, RalphBose, TanimaMistura, LorenzaBoselli, EmanueleMitash, NilayBosnjak, BerislavMithen, RichardBöswald, LindaMithieux, GillesBotelho, Raquel Braz AssuncaoMitola, StefaniaBotella-Carretero, José I.Mitsuishi, YoichiroBott, AlexMiyashita, KazuoBottoms, LindsayMiyata, JunBotturi, AndreaMiyazaki, KoujiBoucher, Barbera J.Mizgier, MałgorzataBoucher, SaraMizuno, AkikoBouillon, RogerMizuno, Cassia S.Bourdon, EmmanuelMizuno, KohBourke, Claire D.Mizuno, TooruBourque, StephaneMizushima, TakashiBoušová, IvaMladěnka, PřemyslBovolin, PatriziaMlejnek, PetrBowers, Laura W.Mobasheri, AliBowling, AprilMoberg, LouiseBowrey, David J.MOCO, SofiaBoy, ErickMóczár, CsabaBoyd, Jacob T.Modica, RobertaBoyer, Justin G.Mohajeri, M. HasanBoyle, Neil BernardMohamed, Azza H.Boysen, ReinhardMohammed Geba, KhaledBozonet, StephanieMohanty, Joy G.Bozso, Sabin J.Moldzio, RudolfBozzetti, FedericoMolendijk, Marc L.Bozzetto, LutgardaMolero Chamizo, AndrésBradshaw, PatrickMolero, Pilar PuertasBraesco, VéroniqueMolina Leyva, AlejandroBragazzi, Nicola LuigiMolina Salinas, Gloria MaríaBrager, AllisonMolinaro, AntonioBragg, MarieMollace, VincenzoBraicu, CorneliaMollard, RebeccaBraig, SimoneMollen, Kevin P.Branca, Jacopo Junio ValerioMøller, GrithBrandao, BrunaMollica, AdrianoBrandão, ElsaMolloy, AnneBrandenburg, Lars OveMomin, ShabamBrantsaeter, Anne LiseMompeo, OlatzBraun, BarryMoncayo, RoyBraun, NicoleMond, JonathanBravo Santos, RafaelMondal, Sujan KBravo-Clemente, LauraMonier, IsabelleBray, GeorgeMonk, JenniferBrecht, MarcusMonks, JeniferBreen, LeighMonnier, VincentBreloy, IsabelleMonroe, CourtneyBremer, PhilMonsen, Anne Lise BjørkeBresciani, LetiziaMontagnani, AndreaBrestoff, Jonathan R.Montalcini, TizianaBretschger Seidelmann, SaraMontaña Blasco, MireiaBreuner, Cora ColletteMonteagudo-Sánchez, CeliaBrewster, LizzyMontecucco, FabrizioBria, EmilioMonteiro, CristinaBriana, Despina D.Monteiro, MarianaBridle-Fitzpatrick, SusanMonteiro-Neto, ValérioBrierley, DanMontejo, Juan CarlosBriggs, MarcMonteleone, PalmieroBrigman, JonathanMontemiglio, LindaBrimblecombe, JulieMontero, EduardoBrink, LaurenMonterosso, John R.Briskey, DavidMontes, NuriaBrito, Maria AlexandraMontgomery, McKaleBritt-Rankin, JoMontoliu-Gaya, LaiaBrivio, PaolaMontone, Carmela MariaBroadhurst, C. LeighMontoro, MiguelBroer, StefanMonzani, AliceBrogi, SimoneMooli, Raja Gopal ReddyBromage, SabriMoon, DamiBromberg, Mark B.Moon, Jong-SeokBronkowska, MonikaMoon, ShinjeBrooks, Alyssa T.Moore, Carolyn E.Brooks, RandallMoore, DanielBrottveit, MargitMoore, Latetia VBrough, LouiseMoore, SallyBroussard, JosianeMorais, Livia HeckeBrouwer, IngeborgMorais, SamanthaBrown Belfort, MandyMorales Suárez-Varela, María M.Brown, FreddyMorales, Francisco J.Brown, HeatherMorales, RodrigoBrown, Robert J.Moran, Nancy E.Brown, Ronald B.Moran-Lev, HadarBrowne, SarahMoranta Mesquida, DavidBrownlee, Iain A.Morante, Juan José HernándezBrownlow, MileneMoreau, RegisBruce, ClintonMorelli, LorenzoBrugiapaglia, AlbertoMoreno Amador, María LourdesBrugliera, LuigiaMoreno Villares, José ManuelBrunaud, LaurentMoreno, Diego A.Bruneau Jr, MichaelMoreno, Jennette PalcicBrunetti, GiacominaMoreno, SilviaBrunetti, LuigiMoreno-Fernandez, JorgeBruno Luigi Rocchi, MarcoMoreno-Indias, IsabelBruns, HeikoMoreno-Maldonado, ConcepciónBruzzese, DarioMoreno-Padilla, MariaBrychta, RobertMoreno-Rojas, José ManuelBryla, PawelMoretti, MassimoBryniarski, KrzysztofMorgan, Emily H.Brytek-Matera, AnnaMorgan, PaulBrzeziński, MichałMorgan-Bathke, Mariabrzoska, malgorzata michalinaMorgese, Maria GraziaBucci, MarcoMori, KatsuhitoBucekova, MarcelaMori, MasaakiBucher Della Torre, SophieMorikawa, ShuntaroBuchet, ReneMorini, GabriellaBuchhorn, ReinerMorino, KatsutaroBücker, RolandMorio, BeatriceBudroni, MarilenaMorisco, FilomenaBudzynska, BarbaraMorisset, Anne-SophieBudzyński, JacekMorita, NaokiBuechler, ChristaMoritz, KarenBueno-Cavanillas, AuroraMoriya, TomoyukiBueno-Lozano, GloriaMorla, ShravanBulla, RobertaMoro, TatianaBullo, ValentinaMoroń, MarcinBunaciu, Rodica P.Morrice, NicolaBunc, VaclavMorrison, Brad E.Bunn, DianeMorrisset, Anne-SophieBunn, JenniferMorseth, Marianne SandsmarkBunt, CraigMoscatelli, FiorenzoBuonocore, DanielaMoseley, KathrynBurak, M.FurkanMoser, OthmarBurek, MalgorzataMoshiri, ArfaBuren, JonasMosoni, LaurentBurge, KathrynMost, JasperBurgess, JohnMoszak, MałgorzataBurgos-Ramos, EmmaMotamed, CyrusBurgstaller, Jakob M.Motherway, Mary O’connellBurkhart, SarahMotlagh Scholle, LeilaBurkhead, Jason L.Motoki, KosukeBurns, DavidMoughan, Paul J.Buro, Acadia W.Moullé, Valentine S.Burrell, LindseyMounien, LourdesBurton, Elvin ThomaseoMounir, BendahmaneBurton, NickMouquet, ClaireBuscemi, JoannaMourino, AntonioBuscemi, SilvioMousa, AyaBusch, MartinMoustaïd-Moussa, NaïmaBusetto, LucaMouterdered, GaëlBusinaro, RitaMoutinho, MiguelBusti, FabianaMovassagh, HesamButaye, PatrickMoyle, LouiseButler, DavidMozas-Moreno, JuanButriss, JudithMozos, IoanaButts, CoryMrazek, AlissaBuxton, SamuelMshvildadze, VakhtangBuzzetti, RafaellaMuccilli, VeraByberg, LiisaMücke, RalphByrne, RebeccaMudgil, PoonamBystrom, JonasMuhle, PaulByun, JaeminMuhlhausler, BeverlyBzikowska-Jura, AgnieszkaMuilwijk, MirtheC Carlson, JennaMukai, RieC. Salvador, LiviaMukherjee, AnubhabCaballero, PabloMukhopadhya, AnindyaCabiscol, ElisaMukhopadhyay, ParthaCabral, CeliaMuldoon, Matthew FrancisCabré, Juan J.Mulier, LanaCacak-Pietrzak, GrażynaMuller, Manfred JamesCaccialanza, RiccardoMüller, MatteaCacciola, FrancescoMüller, ThomasCacho, Nicole TheresaMullie, PatrickCadario, FrancescoMulvihill, ErinCadenas-Sanchez, CristinaMulya, AnnyCádiz-Gurrea, María De La LuzMunday, KarenCadwell, KarinMundi, Manpreet S.Cagnacci, AngeloMunekata, PauloCahill, MichaelMuniraj, NethajiCaiazzo, ElisabettaMuniz Pumares, DanielCaimari, AntoniMuniz, JuanCairo, GaetanoMuñiz, PilarCairo, MontserratMunmany, MeritxellCairrão, ElisaMunoz, Colleen X.Cakebread, Julie A.Muñoz-Alférez, María JoséCalabria, ElisaMuñoz-Durango, KatalinaCalame, WimMuñoz-Galiano, Inés MaríaCalamita, GiuseppeMunshi, KaizadCaldara, Gaetano FeliceMunshi, SoumyabrataCalder, Philip C.Münstedt, KarstenCalderón De La Barca, Ana MaríaMurakami, KosakuCaldovic, LjubicaMurakami, ShigeruCaldwell, JohnMuraru, Iulia-DianaCalle, Mariana CeciliaMurata, ToshihiroCalle-Pascual, Alfonso LuisMurillo-Saich, Jessica D.Callery, Patrick S.Muros, José JoaquínCalon, FrédéricMurotomi, KazutoshiCalvo Lerma, JoaquimMurphy, AliceCalvo, Ana CristinaMurphy, JaneCalvo, MonaMurray, HelenCalvo-Lobo, CésarMurray, KathrynCamara, Amadou K.S.Murray, MargaretCámara-Martos, FernandoMurru, ElisabettaCamarillo, Ignacio G.Murtaugh, MaureenCamejo, GermánMurugaiyah, VikneswaranCamera, DonnyMuruganandan, ShanmugamCamic, Clayton L.Musch, MarkCamier, AuroreMusci, Robert V.Camin, FedericaMuscogiuri, GiovannaCamire, Mary EllenMusilová, ŠárkaCampana, LaraMusolino, VincenzoCampanella, AlessandroMusso, NataleCampanozzi, AngeloMuszyńska, BożenaCampbell, GraceMuszyński, SiemowitCampbell, Sara C.Mutanen, AnnikaCampiche, RemoMuth, Mary K.Campion, BrunoMutoro, Antonina N.Campione, ElenaMuzaffar, HennaCamplain, RickyMuzio, GiulianaCampmans-Kuijpers, Marjo JeMyers, Stephen DavidCampos, DeboraMyhrstad, Mari C.W.Campos, FranciscoMynampati Arunadithya, Bharani KrishnaCanas, JoseMyneni, AjayCanavari, MaurizioMyracle, AngelaCancela, LeonorMysore, YashavanthiCancello, RaffaellaMyszkowska-Ryciak, JoannaCancian, MauroMytych, JenniferCanella, Daniela SilvaNa, MuziCanene-Adams, KirstieNabika, ToruCanepari, MonicaNaciu, Anda MihaelaCannalire, RolandoNadeau, Joseph H.Cano-Ibáñez, NaomiNaderi, SoheilCantorna, MargheritaNadjar, AgnèsCantu-Jungles, ThaisaNagano, TakaoCao, HepingNagaoka, SatoshiCao, Jay J.Nagappan, ArulkumarCao, YingtingNagasawa, YasuyukiCapaldo, BrunellaNagashimada, MayumiCaparrós-González, Rafael ArcángelNagpal, RavinderCaparros-Martin, JoséNagwekar, JanhaviCapasso, RaffaeleNaidu Chitrala, KumaraswamyCapek, Karel D.Naiki, TakuCapel, FredericNair, SaraswathyCapewell, SimonNair, SreenathCapitello, RobertaNair-Shalliker, VisaliniCappelletti, MattiaNajmanová, IvetaCappelli, LucioNakagawa, KenichiroCapper, TessNakagawa, NaokiCapra, SandraNakahama, Ken-ichiCapurso, CristianoNakajima, AtsushiCaputo, MarinaNakajima, HisakazuCaraceni, PaoloNakajima, KeiCaramaschi, DorettaNakajima, KenichiroCarbone, Elvira AnnaNakajima, TamieCarbonel, AnaNakajima, YoshihiroCarbuhn, Aaron F.Nakaki, ToshioCarcea, MarinaNakamura, AkinobuCardenas Zuniga, RobertoNakamura, KensukeCardona, DianaNakamura, KozoCarey, AmandaNakamura, MiekoCariello, MaricaNakamura, TomiyoCarins, JuliaNakamura, TsutomuCarioli, GretaNakano, TakanariCarlberg, CarstenNakao, ReikoCarlberg, JaredNakao, ToshiyukiCarlos, PirolaNakata, MasanoriCarlsen, Monica H.Nakayama, KyosukeCarlson, SusanNakayama, NaomiCarlsson, GunillaNallasamy, PalanisamyCarlsson, MartinNaluai, Åsa TorinssonCarluccio, Maria AnnunziataNamani, TrishoolCarmean, ChristopherNamisaki, TadashiCarmela, NardelliNanno, MasanobuCarmignani, MarcoNanri, HinakoCarmo, IsabelNapierala, MartaCarmona Mejías, RitaNapolitano, AlessandraCarmona-Rivera, CarmeloNarasimhulu, Chandrakala AlugantiCarmona-Torres, Juan ManuelNarayanasamy, SureshCarne, AlanNardelli, CarmelaCarneiro, AngelaNardone, PaolaCarneiro-Barrera, AlmudenaNaruishi, KojiCarobbio, StefaniaNascimento, MarcusCarpenter, ElizabethNascimento, Weslania VivianeCarpenter, GuyNasef, NohaCarper, MichaelNaser, Abu MohdCarpi, SaraNashine, SonaliCarr, Loneke BlackmanNasiri Ansari, NarjesCarr, William H.Natarajan, Sathish KumarCarra, Maria ClotildeNath, ArijitCarrard, IsabelleNathubhai, AmitCarrasquilla, GermanNaughton, RobertCarriazo, SolNaughton, ShaanCarrie, GrueterNaumann, ElkeCarriker, ColinNaumann, MichaelCarrillo-Álvarez, ElenaNavarra, MicheleCarroll, Harriet A.Navarrete-Munoz, Eva MariaCarson, BrianNavarro Flores, EmmanuelCaruso, GiuliaNavarro, CasildaCarvajal, MaríaNavarro, EstanisCarvelli, LuciaNavarro, Sandi L.Casaer, Michael P.Navarro, VirginiaCasagrande Figueiredo, VandréNavarro-Alarcon, MiguelCasals, GregoriNavarro-Flores, EmmanuelCasanova, Alfredo GinésNavarro-Pérez, Carmen F.Casanova, LlanosNavas-López, Víctor ManuelCasanovas, CarmenNayanatara, Arun KumarCasanueva, Felipe F.Nazare, Julie-AnneCasas, Rosa M.Nazzaro, GianlucaCasella, GiovanniNdrepepa, GjinCasey, Theresa M.Neag, Maria AdrianaCasirati, AmandaNeff, RoniCasperson, Shanon L.Negi, VinnyCassard-Doulcier, Anne MarieNegri, EvaCassidy, OmniNegro, MassimoCassidy, SophieNeilson, AndrewCassisi, Jeffrey E.Nejati, FatemehCastagna, MichelaNelson, AlexandraCastaldo, GiuseppeNelson, DavidCastano-Vinyals, GemmaNelson, GradyCastellani, ChristophNelson, GreggCastelli, LuciaNelson, RolfCastelló, AdelaNenna, AntonioCastiglione, RobertoNepal, Mahesh RajCastillo-Fernandez, JuanNeri, CaterinaCastonguay, JessicaNescolarde, LexaCastren, MaijaNestares Pleguezuelo, Maria TeresaCastresana, Javier S.Neubauer, KatarzynaCastro, IanaNeves, JoãoCastro, Juan C.Nevola, RiccardoCastro-Sepulveda, MauricioNevzorova, Yulia A.Casu, CarlaNewell, MarnieCatalano Iniesta, LeonardoNewell-Fugate, Annie E.Catalano, AntoninoNewmire, DanielCatanzaro, RobertoNewton, RobertCatar, RusanNexo, EbbaCattaneo, CamillaNexø, EbbaCatto-Smith, AnthonyNeymotin, FlorenceCatucci, GianlucaNg, Hooi HooiCaulfield, LauraNg, Shu WenCautela, DomenicoNguo, KayCavaliere, GinaNguyen, Long TheCavallarin, LauraNguyen, TuCawthon, Peggy MannenNgwube, AlexanderCazals-Hatem, DominiqueNi Lochlainn, MaryCazzaniga, AlessandraNicastro, Holly L.Cazzola, RobertaNichenametla, Sailendra N.Cè, EmilianoNicholl, AnaliseCebey-López, MiriamNichols, Buford L.Cecchetti, SerenaNichols, Robert G.Ceci, Luigi R.Nickerson, BrettCederberg, ChristelNicolaides, StevenCedó, LídiaNicoletti, ClaudioCelano, GiuseppeNie, PengCelebioglu, Hasan UfukNiedzwiedzka, EwaCeliberto, LarissaNielsen, DaviaCelie, BertNielsen, Forrest HaroldCelis-Regalado, Luis G.Nielsen, TrineCena, HellasNiemi, Anna KaisaCensi, SimonaNiemiro, Grace M.Centanni, MarcoNieoczym, DorotaCeolotto, GiulioNier, AnikaCeresoli, MarcoNieradko-Iwanicka, BarbaraCerletti, ChiaraNieri, PaolaCermeno, MariaNieuwoudt, StephanCervantes, JorgeNighot, PrashantCesareo, RobertoNii, MasafumiCetin, IreneNiinimäki, MaaritCetrullo, SilviaNikawa, TakeshiCeulemans, DriesNiknamian, SorushCha, Hwa JunNiles, Richard MChaaban, HalaNilsen, Roy M.Chace, Donald H.Nilson, Eduardo A. F.Chacin-Suarez, AudryNilsson, AkeChagoury, OdetteNilsson, AndreasChakaroun, Rima M.Nilsson, IdaChakrabarti, PriyadarshiniNinomiya, KiyofumiChakrabarti, SubhadeepNishi, MayumiChalari, EleannaNishi, Stephanie K.Chalifour, LorraineNishida, KeigoChallamel, GhislaineNishijima, ChiharuChamaria, SurbhiNishikawa, TomofumiChamberland, JulienNishimura, NorihiroChambers, Joseph E.Nishioka, ShintaChamcheu, Jean ChristopherNishizuka, MakotoChamitava, LiliyaNissen, LorenzoChamoun, ElieNissinen, Markku J.Chamulitrat, WaleeNiu, TianhuaChamut, SteffanyNixon, Daniel W.Chan, CatherineNlandu, Roger NgatuChan, LungNobile, StefanoChan, Marion M.Noel, Sabrina E.Chan, Yin-ChingNoel, SanjeevChandler, Paulette DNofer, Jerzy-RochChandrababu, RameshNoga, MarekChandran, VinodNogueira, Rossana C.Chandrasekaran, KrishNoguera Artiaga, LuisChang, Chih-HsiangNohara, KazunariChang, Ching-ChihNohr, DonatusChang, EdwinNolan, JohnChang, EugeneNolden, AlissaChang, Fu-KueiNoll, MatiasChang, Hsin-I.Nomikos, TzortzisChang, Hui-HuaNomura, KyokoChang, Jer-MingNomura, YasuhiroChang, Kee-LungNorambuena, FernandoChang, Ke-VinNoratto, GiulianaChang, Mei-WeiNordestgaard, Ask TybjærgChang, Nai-JenNordhagen, StellaChang, Shu-ChenNorsa, LorenzoChang, Te-ShengNorte Navarro, Aurora IsabelChang, Yen-ChangNorton, CatherineChantler, PaulNorval, MaryChao, Chia-TerNorwitz, NicholasChao, Hsu-WenNot, TarcisioChao, Pi-YuNotarnicola, MariaChaplin, AliceNovak, FrantisekCharlton, ClivelNovak, SanjaCharoensiddhi, SuvimolNowacka-Jechalke, NataliaChase, P. BryantNowak, Elizabeth CChatani, MasahiroNowakowska-Zajdel, EwaChatel, BenjaminNowicka, GrażynaChaurasia, BhagirathNowotarski, Shannon L.Chauveau, ChristopheNtemiri, AlexandraChavanelle, VivienNualart, FranciscoChavatte, LaurentNuessler, AndreasChaves-Martinez, Felipe JavierNugent, AnneChavez-Santoscoy, Rocio AlejandraNunes, CarlaChazot, CharlesNunes, Everson A.Checchi, MartaNunes, FernandoChee, MelissaNunnery, DanielleCheema, SukhinderNurwidya, FarizCheemarla, NagarjunaNuthikattu, SaivageethiChelikani, Prasanth K.Nuutila, PirjoChen, Chiao-MingNuzzo, DomenicoChen, Chih-YenNyangahu, DonaldChen, Chi-NienO’Brien, Cecelia M.Chen, Chiung-meiO’Brien, Kimberly O.Chen, Chung-HwanO’Brien, Sarah H.Chen, Chung-Yen OliverO’Connell, Amy E.Chen, DanhongO’Connor, LaurenChen, Der-YuanO’Donnell, MartinChen, DongbaoO’Leary, FionaChen, Fang-PeiO’Sullivan, AifricChen, GuangpingO’Sullivan, MariaChen, HongO’Sullivan, OrlaChen, Hsin-JenOakey, ZackeryChen, Jen-YinOba, ShinoChen, Jiann-ChuObana, AkiraChen, Jin-ShuenObanda, DianaChen, Kong Y.Obeid, OmarChen, Kuan-FuObeid, RimaChen, Kuo-HuOberhelman, SaraChen, LiObermayer-Pietsch, BarbaraChen, Li-HanObling, Sine RoelsgaardChen, LiliObri, ArnaudChen, LinOcaña, María Del CarmenChen, Li-ShengOchiai, HirokoChen, Pao-HuanOchoa, JulioChen, QiOcké, MargaChen, Rong-JaneOczkowski, MichałChen, Sheng-HungOda, MasatakaChen, Shuen-EiOda, YoichiroChen, Szu-ChiaOda, YoshiyaChen, Vincent C.W.Odamaki, ToshitakaChen, Vincent L.Odrowaz-Sypniewska, GrażynaChen, WeiOest, Megan E.Chen, WeiqinOgasawara, RikiChen, XinhuaOgawa, MitsutakaChen, Xuqi (Ricky)Ogawa, YouichiChen, Ya-LingOgbourne, Steven M.Chen, Yen-PoOgino, ShujiChen, Yi ChunOgrodowczyk, AnnaChen, Yi-FanOgunjirin, AdebowaleChen, YiliangOh, ByungchulChen, Yi-MingOh, Deog-HwanChen, Yu-ChiehOh, Kook-HwanChen, Yue-HwaOh, Seung HyunChen, Yue-WenOh, SooyoungChen, Yu-JenOh, Tae JungChen, Yun-WenOhira, AkihiroChendrasekhar, AkellaOhla, KathrinCheng, HelenOhlson, BodilCheng, Hong ShengOhnishi, ShihoCheng, Juei-TangOhno, JunCheng, Pei-WenOhrenberger, GeraldCheng, Tuck SengOhta, TakahisaCheng, Xian-wuOjima, KoichiChenoll, EmparOjo, OmorogievaCherkaoui Malki, MustaphaOkada, HiroshiCheungpasitporn, WisitOkada, KohkiChhetri, Jagadish K.Okada, ShinjiChiang, Chih-PoOkada, TomoyoChiang, Meng-TsanOkamura, TakuroChiappetta, CaterinaOkano, YoshiyukiChiarioni, GiuseppeOkayama, MasanobuChiba, HitoshiOkuda, MasayukiChichger, HavoviOkuda, NagakoChicoine, BrianOkumura, ToshikatsuChiechio, SantinaOlarte-Mantilla, SandraChiellini, GraziaOlaru, Octavian TudorelChien, Chun-RuOlayanju, Julia B.Chien, Li-YinOlchowik-Grabarek, EwaChien, Yi-wenOldewage-Theron, WilnaChilibeck, PhilOlea Serrano, NicolásChim, Shek ManOlech, MartaChimin, PatriciaOledzka, GabrielaChin, Kok YongOlendzki, BarbaraChin, Young-WonOlišarová, VěraChing, Tsui-TingOliveira, Maria ManuelChinnakannan, SenthilOlivier-Van Stichelen, StéphanieChiou, Ya-LingOllberding, Nicholas J.Chitchumroonchokchai, ChureepornOlmedillas, HugoChitraju, ChandramohanOlmedo-Requena, RocíoChiu, Kuo-HsunOlomu, IsokenChiu, Ping-FingOlsen, Nanna JulieChiu, SimonOlsen, ThomasChiu, Wan-ChunOlum, SolomonChiu, Ya-HuiOmagari, KatsuhisaChlíbková, DanielaOmar, Syed HarisCho, ClaraOmidian, KosarCho, Nicole A.Onieva-Zafra, María DoloresCho, SungeunOniszczuk, AnnaChoi, DonghoÖnning, GunillaChoi, Eung HoOnnis, ValentinaChoi, Huimahn A.Onusic, SylviaChoi, Hyung JinOnyenwoke, Rob U.Choi, Hyung-KyoonOoi, Chee Y.Choi, InwookOpata, MichaelChoi, Jeong JuneOpere, Catherine A.Choi, Jeong-HwaOpoku-Acheampong, AudreyChoi, Je-YongOpydo-Szymaczek, JustynaChoi, Jin WooOralová, VeronikaChoi, JinkyungOrczyk-Pawiłowicz, MagdalenaChoi, Soo JeongOredsson, StinaChoi, Yung-HyunOrellana, EstebanChojnacki, JanOrenes-Piñero, EstebanChong, Cher-RinOrf, IsabelChoromanska, AnnaOrlandini, MaurizioChou, Yu-ChingOrlando, AntonellaChouinard-Watkins, RaphaëlOrlemann, TillChoukri, MariaOrsi, LuigiChoung, Se YoungOrtega, ÁngelesChouraqui, Jean-PierreOrtega-Calvo, ManuelChow, Lisa S.Ortiz, AndricChowdhury, SabihaOrttung, Robert W.Chreptowicz, KarolinaOsada, JesúsChristakoudi, SofiaOsborne, John O.Christian, FrancisOse, JenniferChristians, JulianOsella, Alberto RubénChristides, TatianaOsganian, Stavroula K.Christodoulou, Maria-IoannaOshima, ShunjiChristophersen, ClausOssoli, AliceChtourou, HamdiÖstberg, Anna-LenaChu, Pei-YiÖstenson, Claes-GöranChu, Sang HuiOster, MichaelChun, Ock K.Ostrowska, LucynaChung, Hae-YoungOthman, Eman M.Chung, Ming YanOtsuka, IsaoChung, RosannaOtsuka, YuzuruChung, Yessica C.Y.Ouwehand, ArthurChurch, DavidOvercash, Francine M.Church, TimothyOwari, YutakaChutiyami, MuhammadOwczarek, AleksandraChuyen, Hoang V.Ozaki, C. KeithChycki, JakubOzawa, YokoCiacci, CarolinaOzodiegwu, IfeomaCianciulli, AntoniaOzturk, Orgul D.Ciapaite, JolitaPabisiak, KrzysztofCiappolino, ValentinaPabón-Carrasco, ManuelCiardullo, StefanoPabst, AndreasCibelli, GiuseppePace, PaulCibulskis, CatherinePaczkowska-Abdulsalam, MagdalenaCicco, Paola DePadhi, EmilyCiccone, Marco MatteoPadilha, MarinaCicerale, SaraPadilla, NathaliaCicero, ArrigoPadilla-Benavides, TeresitaCiclitira, PaulPadmanabhan, JagannathCid Canda, María ConcepciónPadmanabhan, VasanthaCiebiera, MichalPádua, InêsCieślińska, AnnaPaepegaey, Anne-CécileCifelli, Christopher J.Pafundi, Pia ClaraCimmino, LuisaPagano, AlessandraCinelli, GiuliaPagano, GiovanniCingolani, AriannaPagano, LoredanaCintoni, MarcoPage, AmandaCioffi, FedericaPaik, Hyun-DongCioffi, IolandaPaik, JisunCione, ErikaPainter, Jodie N.Cipriani, CristianaPajkrt, EvaCirino, PatrickPal Choudhuri, ShreoshiCirulli, FrancescaPalanisamy, Uma DeviCiuoli, CristinaPalanski, BradCladis, DennisPalau, PatriciaClanchy, FelixPalazón-Bru, AntonioClark, SeanPalermo, FrancescoClarke, NeilPali-Schöll, IsabellaClaro, CarmenPalko-Labuz, AnnaClaro, Rafael MoreiraPalladini, GiuseppinaClassen, Hans-GeorgPallafacchina, GiorgiaClaude, BilleaudPallàs Lliberia, MercèClaus, PeterPallauf, KathrinClayton, Zachary S.Pallottini, ValentinaClemens, Robert K.Palmeira, CarlosClemens, RogerPalmela, CarolinaClemente-Suárez, Vicente JavierPalmer, ChristopherCliff, Margaret A.Palmer, DebraClifford, TomPalmer, RaymondClugston, RobinPalmerius, Karljohan LundinClulow, AndrewPalmieri, OrazioCoan, P. M.Palomba, LetiziaCoates, PenelopePalombi, LeonardoCobbold, Peter H.Palsamy, PeriyasamyCobelens, FrankPalumaa, PeepCockburn, DarrellPalumbo, Vincenzo DavideCodella, RobertoPamir, NathalieCoe, Christopher L.Pamoukdjian, FredericCoelho, AnaPan, FengCoelho, TracyPan, Hung-ChuanCoenders, GermàPan, XiaoyueCohen, JulianaPan, YijunCohen, Michael S.Pan, Yuan-XiangCohen, RonaldPanda, SivaCoker, Robert H.Pande Katare, DeepshikhaColaizzo-Anas, TinaPandolfi, AssuntaColantuono, AntonioPanek, DawidColavita, GiampaoloPanek-Shirley, Leah MCole, MarshaPangrazzi, LucaColenbrander, AugustPanico, AlessandraColetti, DarioPanizza, ChloeCollado, Pilar SánchezPankratova, StanislavaColleluori, GeorgiaPannala, AnanthCollins, AdamPanos, Georgios D.Collins, FraserPantano, Kathleen JoanCollins, James F.Pantea Stoian, AncaColmán Martínez, MarielPantham, PriyadarshiniColombo, JohnPanwalkar, PoojaColquhoun, RyanPanza, RaffaellaColson, NataliePanzuto, FrancescoColtell Simón, OscarPaoletti, Anna MariaColtell, OscarPaolucci, MatteoColucci, RocchinaPapachristodoulou, AlexandrosCombaret, LydiePapada, EfstathiaComeche, Jose M.Papadaki, AngelikiComelli, ElenaPapadimitriou, KonstantinosCommane, DanielPapadopoulos, DimitriosCOMSA, ElisabetaPapadopoulou, SousanaComstock, SarahPapadopoulou-Legbelou, KyriakiComulada, Warren ScottPapamichail, KonstantinosConde, SilviaPapanastasiou, AnastasiosConlon, MichaelPapanikolaou, EleniConnaughton, Victoria P.Papanikolaou, YanniConner, Tamlin S.Papapanagiotou, VasileiosConrad, ZachPapathakis, Peggy CallaghanConroy, DeirdrePapathanasiou, EvangelosConstantin-Teodosiu, DumitruPapet, IsabelleConte, ElenaPapi, AlessioConte, GiuseppePaplinska, MagdalenaContreras, CristinaParackal, SherlyConway, MyraParadiso, AngeloCooke, MatthewParajuli, RajendraCooper, JamieParazzini, FabioCopeland, PaulParekh, NiyatiCoppola, AndreaParhofer, Klaus G.Coppola, GianlucaParis, JasonCoras, RoxanaParisi, LudovicaCorbin, Karen D.Park, ChloeCordaro, MarikaPark, Dong SunCordera, RenzoPark, Hun-YoungCordes, TheklaPark, InkyuCordido, FernandoPark, Jung TakCórdova, AlfredoPark, Kyoung-ChanCorica, FrancescoPark, KyoungminCorish, ClarePark, Min JungCornel, MartinaPark, Phil JuneCornish, M. LynnPark, Sang WonCornish, StephenPark, Se JinCornu, CatherinePark, Si HongCorpe, ChristopherPark, Soo JeanCorrea, Diego AlzatePark, Sook-HyunCorrea-Rodríguez, MariaPark, YongsoonCorsello, Salvatore MariaPark, Yoo KyoungCorti, ChiaraParkar, Shanthi G.Coscia, AlessandraParker, Elizabeth A.Cosín-Roger, JesúsParker, HelenCosín-Tomàs, MartaParker, Leslie AnnCosta Ribeiro, Cecilia ClaudiaParker, MeganCosta, Maria Do CéuParlesak, AlexandrCosta, MatheusParmar, AdityaCosta, Pablo B.Parnell, JillCosta, RaquelParodi, EmiliaCostabile, GiuseppinaParoni, RitaCosta-Izurdiaga, AliciaParrasia, SofiaCostantini, LaraParrett, AlisonCostanzo, SimonaParrettini, SaraCottin, SarahParrott, Julie M.Cottone, PietroParry-Strong, AmberCouce, María LuzParsanathan, RajeshCoulson, SamanthaParson, Simon H.Coussons, PeterParsons, LindaCovasa, MihaiPartridge, StephanieCovello, GiuseppinaPartyka, AnnaCovert, Hannah H.Pasanisi, FabrizioCowan, CaitlinPasca, LudovicaCowardin, CarriePascal, LauraCox Sullivan, SheilaPaschalis, VassilisCox, AmandaPasini, LuigiCox, Christopher R.Paslakis, GeorgiosCox, Natalie J.Passos, Cláudia P.Coyle, Edward F.Pastor, RosarioCózar-Castellano, IrenePastor-Valero, MariaCramer, ThorstenPastor-Villaescusa, BelénCrapo, CharlesPatankar, Jay V.Crasta, JewelPatarrão, RitaCraveiro, DanielaPatel, Barkha P.Creasy, SethPatel, ChloeCrehuá-Gaudiza, ElenaPatel, Daxesh P.Cremon, CesarePatel, DevenCrenn, PascalPatel, JenilCrescenzo, RaffaellaPatel, UrvishCresci, Gail A.Paterniti, IreneCrespo-Escobar, PaulaPathak, DhrubaCrestani, MaurizioPatik, JoradanCrichton, Robert R.Patricia, Concheiro-MoscosoCrimarco, AnthonyPatro-Małysza, JolantaCrispin, PhilipPatruno, AntoniaCrosby, KarenPatruno, CataldoCroset, MartinePatruno, MarcoCrosetto, PaoloPatterson, CarsonCross, Carroll E.Patterson, EmmaCrowe, KellyPatterson, Mindy A.Cruz-Hernandez, CristinaPaudel, BishalCuadrado-Cenzual, Maria AngelesPaul, AlokCuesta, DanielPaul, AntoniCuffaro, DorettaPaul, ManoranjanCuffe, JamesPaul, Siba ProsadCukrowska, BoženaPaul, SudipCuletu, AlinaPauling, JoschCumming, Kristoffer ToldnesPaulo Mirasol, EstherCummins, AdrianPaulsen, Mari MohnCunningham, AmyPaventi, GianlucaCupisti, AdamascoPavlides, MichaelCurci, ClaudiaPavlov, EvgenyCurtelin, DavidPavone, PieroCurtis, Peter J.Pawlaczyk, AleksandraCurto, AdrianPawlak, DariuszCussotto, SofiaPayo, Rubén MartínCutone, AntimoPaz Montelongo, SorayaCygankiewicz, AdamPazdro, RobertCzaja, KrzysztofPeacock, CoreyCzaja-Bulsa, GrażynaPearl, Rebecca L.Czarniecka-Skubina, EwaPeck, Dawn S.Czenczek-Lewandowska, EwelinaPedersen, Kim B.Czepczor-Bernat, KamilaPei, YonggangCzerwinska, Areta MagdaPeila, ChiaraCzerwińska, MonikaPeirang, CaoCzerwonogrodzka-Senczyna, AnetaPeiris, MadushaCzlapka-Matyasik, MagdalenaPekkala, SatuCzogalla, KatrinPelczyńska, MartaCzuba, MiłoszPeleg-Raibstein, DariaCzuba, ZenonPelidou, Sygkliti-HenriettaCzumaj, AleksandraPellas, NikolaosCzyż, KatarzynaPellegrini, MarikaCzyż, StanisławPellegrini, NicolettaD’Aloisio, RossellaPellicano, RinaldoD’Angelo, StefaniaPellizzon, Michael A.D’Archivio, MassimoPeña Quintana, LuisD’auria, EnzaPena, MariaD’avolio, AntonioPena, Maria MarjoretteD’Elia, LanfrancoPeña, SebastiánD’Souza, Randall FelixPencharz, Paul B.Da Silva Dias, Igor AlexandrePenfold, ChristopherDąbrowski, MariuszPeng, MeiDachs, GabiPeng, YangDaci, ArmondPeng, Yi-JenDacrema, MarcoPenna, FabioDaguet, DavidPennington, Kathleen A.Dahdah, LamiaPenniston, KristinaDahl, LisbethPenno, MeganDahl, WendyPenumarti, AnushaDai, ZhaoliPenzol, María JoséDaidone, MarioPepe, JessicaDai-Keller, JoyPepino, M. YaninaDal Ben, MatteoPerakakis, NikolaosDall’Asta, MargheritaPeraldi, PascalDallacker, MatteaPerandini, Luiz AugustoDallio, MarcelloPercival, Benita C.Dalrymple, Kathryn V.Percival, Susan S.Daly, AlisonPerdomo, GermanDaly, AnnePereira Machado, PriscilaDambrauskas, ZilvinasPereira, Carlos DiasDamiani, GiovanniPereira, Cidália AlmeidaDamiano, FabrizioPereira, Cláudia Maria F.Damoiseaux, JanPereira, SóniaDandekar, AdityaPereira-da-Silva, LuísDando, RobinPereiro, RosarioDanesi, FrancescaPerera, Conrad O.Daniele, AntonellaPeretti, NoëlDaniele, AuroraPérez Marfil, Maria NievesDanielewicz, AnnaPerez Vizcaino, FranciscoDaniluk, UrszulaPérez, SalvadorDar, Mohd SaleemPérez-Álvarez, Jose AngelDaragó, AdamPérez-Bosque, AnnaDarcy, JustinPérez-Burillo, SergioDarmaun, DominiquePerez-Cano, FranciscoDarwish, AmiraPérez-Gálvez, AntonioDas, AbhirupPérez-Jiménez, JaraDas, AditiPérez-Lopez, AlbertoDas, Anibh M.Pérez-López, AlbertoDas, NupurPerez-Martinez, PabloDas, SadhanPérez-Ortiz, José M.Datta, PoulamiPérez-Rodrigo, CarmenDattola, AnnunziataPérez-Ros, PilarDave, Jayna M.Periasamy, RameshDavey, AdamPerissiou, MariaDavid, Cameron-SmithPerna, SimoneDavidson, LancePeron, GregorioDavis, CourtneyPerrin, Maryanne T.Davis, David E.Perrotta, AdolfoDavis, Dustin W.Perry, Cydne A.Davis, KathleenPerry, NicoletteDavison, GlenPersechino, SeverinoDawson, CharlottePerticone, FrancescoDay, RichardPerticone, MariaDe Amicis, RamonaPerucha, EsperanzaDe Backer, GuyPervin, MoniraDe Bock, Geertruida HendrikaPeschel, Anne O.De Boer, AliePessi, TanjaDe Boni, AnnalisaPeters, John C.De Castro, GabrielaPetersen, KristinaDe Chiara, FrancescoPeterson, Christine TaraDe Clercq, Nicolien C.Peterson, CourtneyDe Cock, DiederikPeterson, JuliaDe Filippis, AnnaPetito, AnnamariaDe Filippo, GianpaoloPetropoulos, IsabelleDe Frutos, Pablo GarcíaPetrov, Maxim S.De Groot, EricPetrova, AnnaDe Groot, Lynette M.Petta, IoannaDe Guia, Roldan M.Pettersen, Jacqueline A.De Kreutzenberg, SaulaPetzer, VerenaDe La Monte, SuzannePezeshki, AdelDe La Parra, ColumbaPezzotto, Stella MarisDe La Rubia Ortí, José EnriquePfeiffer, AndreasDe La Torre, RaphaelePham, ThaoDe Lange, PieterPhan, Chin-SoonDe Las Heras Montero, Javier AdolfoPhan, Minh Anh ThuDe Las Heras, JavierPhang, JamesDe Leeuw, Peter W.Pharaoh, GavinDe Leo, VincenzoPhelps, Kenneth R.De Leon, AngelaPhilips, ThomasDe Los Angeles Serradell, MaríaPhillips, Mary E.De Luca, PasqualePhinney, StephenDe Lucia, ClaudioPiacentini, Maria FrancescaDe Man, Joris G.Piancatelli, DanielaDe Martin, SaraPiantoni, SilviaDe Martinis, MassimoPiasecki, MathewDe Matteis, AlessandraPiatek, JacekDe Mendonça, Dina I. M. D.Piątkowska, EwaDe Moura, Ana PintoPicaud, Jean-CharlesDe Paco Matallana, KatyPicchianti-Diamanti, AndreaDe Pascual-Teresa, SoniaPiccinin, ElenaDe Pergola, GiovanniPiccirillo, RosannaDe Rosa, VivianaPiccoli, GiorginaDe Sanctis, Juan BautistaPiccolo, MarialuisaDe Simone, ClaudioPickova, JanaDe Sire, AlessandroPickrell, AliciaDe Sire, RobertoPicó-Monllor, José AntonioDe Smet, StefaanPiecha, GrzegorzDe Sotomayor, Maria AlvarezPieczyńska, JoannaDe Souza Santos, RobertaPiekara, AgnieszkaDe Vadder, FilipePiemontese, LucaDe Vecchis, RenatoPiemontese, PasquaDe Vogel-Van Den Bosch, JohanPiepenbrink, KurtDe Vos, PaulPierce, GrantDe Vries, JanPierre-Antoine, DuguéDe Wet, HeidiPierri, LucaDe Zwaan, MartinaPieszka, MagdalenaDean, ElizabethPietrangelo, TizianaDearden, LauraPietruszewska, WiolettaDeb, SubrataPietruszka, BarbaraDe-Bandt, Jean-PascalPifferi, FabienDebora, Barnes-JosiahPigłowska, MałgorzataDeBosch, BrianPiia, SimonenDebourdeau, Philippe M.Piknova, BarboraDebruin, NatasjaPilecky, MatthiasDedoussis, George V.Pileggi, ChantalDeehan, Edward C.Pilkington, Suzanne M.Defoort, CatherinePilla, RaffaeleDeierlein, Andrea L.Pillon, Nicolas J.Dekker, MarloesPilutin, AnnaDekker, Peter J. T.Pilz, StefanDel Ben, MariaPimenta, Nuno M.Del Bo, CristianPimiento, Jose M.Del Giorno, RosariaPinacchio, ClaudiaDel Pinto, RitaPinel, AlexandreDel Pozo De La Calle, SusanaPinel, KarineDel Rio, DanielePinho, Maria Gabriela M.Delacour, HervéPinkas, AdiDelaissé, Jean-MariePinto, AnaDelarue, JacquesPinto, ElisabeteDelaune, VaiherePinto, LorisDelcour, JanPinto-Sanchez, Maria InesDeleye, YannPiotrowska, KatarzynaDelhanty, PatricPippi, RobertoDeli, Chariklia K.Pirastu, NicolaDelimont, NicolePirola, LucianoDelisle, HélènePisani, Didier F.Delitala, AlessandroPisani, LucianaDeliyanti, DevyPiska, KamilDella Bella, ElenaPittaluga, AnnaDella Morte, DavidPitti, Julio AlvarezDella Pepa, GiuseppePittler, Steven J.Delmonico, Matthew J.Pitucha, GrzegorzDeluccia, RosemaryPizzoferrato, MarcoDelvin, EdgardPlacek, CaitlynDe-Magistris, TizianaPlaisance, Eric PaulDemaison, LucPlastina, PierluigiDemarquoy, JeanPlaza-Díaz, JulioDemchenko, Alexei V.Pletsch-Borba, LauraDeme, PragneyaPlevinsky, JillDemers-Mathieu, VeroniquePlichta, MartaDemmelmair, HansPlösch, TorstenDen Braber, NialaPluijm, SaskiaDen Hartigh, Laura J.Plummer, SueDenford, SarahPobee, Ruth AdisetuDengler, FranziskaPochi, SubbarayanDenkova-Kostova, Rositsa StefanovaPoff, AngelaDepalma, Ralph G.Pohl, PawelDepeint, FlorePohlig, Ryan T.Deptuła, MilenaPohlkamp, TheresaDerave, WimPokorski, MieczyslawDerbyshire, EmmaPolakof, SergioDereń, KatarzynaPolanska, KingaDeriu, FrancaPolesel, JerryDesai, Ajay S.Poli, AndreaDesai, JigarPoli, GuiseppeDesai, MinaPolis, BaruhDesai, NiyatiPolishchuk, RomanDeschasaux, MelaniePolito, RitaDeshpande, GauraviPolizzi, ElisabettaDesiderio, AntonellaPolverino De Laureto, PatriziaDesjarlais, MichelPons-Duran, ClaraDesselberger, UlrichPontonio, EricaDeth, RichardPontzer, HermanDetopoulou, ParaskeuiPoobalan, AmudhaDettweiler, UlrichPopelová, AndreaDetzel, PatrickPopiołek-Kalisz, JoannaDeutsch, MelaniePopkin, BarryDeutz, Nicolaas E.P.Popova, Inna E.Devabhaktuni, Subodh R.Popp, AlinaDevarshi, Prasad P.Porat, DanielDevine Eller, AudreyPorebski, GrzegorzDevkota, SuzannePorta, Matteo DellaDevlieger, RolandPortella, AndreDey, Amit KumarPortero, ManuelDey, PriyankarPosadino, Anna MariaDezaki, KatsuyaPoso, AnttiDhombres, FerdinandPosovszky, CarstenDi Baldassarre, AngelaPostler, Thomas S.Di Daniele, NicolaPot, GerdaDi Felice, ValentinaPotaczek, Daniel P.Di Francesco, SimonaPotenza, Maria AssuntaDi Giacomo, SilviaPothos, Emmanuel N.Di Lorenzo, CherubinoPotter, MichaelDi Lorenzo, ChiaraPotter, MurrayDi Maso, MatteoPoudel, Sher BahadurDi Mauro, MariaPourafshar, ShirinDi Nunzio, MattiaPourová, JanaDi Paola, RosannaPourzand, ChararehDi Pasqua, Laura GiuseppinaPouteau, EtienneDi Pierro, ElenaPowell, RebeccaDi Pietro, PaolaPowell, TheresaDi Renzo, LauraPower, KristaDi Rienzi, SaraPowers, JohnDi Salle, AnnaPowrózek, TomaszDi Stadio, AriannaPozzi, MarcoDi Stefano, RossellaPozzobon, MichelaDi Vita, GiuseppePrabhu, Antony HeroldDiamond, GillPrada, MaríliaDiane Valeriano, ValeriePraktiknjo, MichaelDiaz, Eva C.Prasad, Kedar N.Díaz, Juan JoséPrats, María SoledadDíaz-Castro, JavierPratsinis, Sotiris E.Dibaba, DanielPravst, IgorDibaba, Daniel T.Prawitt, JanneDiBattista, AliciaPremawardhana, LakdasaDibay, SepidehPremilovac, DinoDickerson, RolandPrendergast, LukeDiehl, KatharinaPrescha, AnnaDiehl, LindaPrescott, MelissaDietrich, Johannes W.Pries, Alissa M.Díez López, IgnacioPrieto, IsabelDiez-Sampedro, AnaPrieto, Rafel M.Dima, AlinaPrieto-Garcia, JoséDimitrellou, DimitraPrieto-Lloret, JesusDimitriadis, GeorgiosPrimeaux, StefanyDina, RăzvanPrincipia, Scavo MariaDinas, PetrosPrinz, PhilipDinh, Quynh NhuPritchett, KellyDinis-Oliveira, Ricardo JorgePriyadarshini, AnushreeDioguardi, Francesco S.Priyadarshini, MedhaDiou, ChristosProbert, ChrisDioum, El Hadji M.Prochazkova, PetraDireito, RosaProcter, Sandra B.DiSilvestro, RobertProdam, FlaviaDivakaruni, SrinivasProietto, JosephDivino-Filho, JosèProkesch, AndreasDix, ClareProtogerou, Athanase D.Dixon, HelenProtudjer, JenniferDjuric, ZoraProudfoot, DianeDludla, Phiwayinkosi V.Prowans, PiotrDłuski, DominikPrzekora, AgataDo Carmo, SoniaPrzybylowicz, KatarzynaDobenecker, BrittaPrzybylska-Balcerek, AnnaDobrowolski, HubertPuchades, María JesúsDocasar, Edurne ÚbedaPuddu, Paolo EmilioDocherty, Neil G.Puell, Maria CintaDodge, ElizabethPuga, Ana M.Doering, Thomas M.Pugh, JamieDoerr, CelestePugin, BenoitDoherty, RónánPugni, LorenzaDolgopolova, IrinaPujia, ArturoDoligalska, MariaPulido, OlgaDolin, Cara D.Pulker, ClaireDomenicotti, CinziaPullar, JulietDomínguez Díaz, LauraPulle, Ranjeev ChrysanthDominguez, LigiaPullen, NicholasDomínguez-Vías, GermánPunchard, Neville ADominika, Jamioł-MilcPundir, ShikhaDonadel, GiuliaPuppa, MelissaDonato, CarmelaPurcell, Sarah A.Donato, RosarioPurdom, TroyDong, FengPursey, KirrillyDong, Huan-JiPüschel, Gerhard P.Dong, KellyPutot, AlainDong, Tien SyPutta, AnjaneyuluDonniacuo, MariaPycia, KarolinaDonofry, ShannonPyne, DavidDonohue, Lee T.Pyter, Leah M.Donovan, Sharon M.Pyzik, MichalDordevic, AimeeQin, LuyeDorling, JamesQiu, BinDorris, Emma R.Quadros, EdwardDörsam, Annica F.Quartu, MarinaDorta, EvaQuigley, EamonnDos Santos, Klinsmann CaroloQuiles, JoanDoseff, Andrea I.Quintero-Florez, AngelicaDoshi, SimitQuist, Jonas SallingDou, QingpingR. Appling, DeanDougkas, AnestisR. DeFelice, AmyDoulberis, MichaelRabinowitz, Joshua D.Dounousi, Evangelia C.Racette, LyneDovnik, AndrazRachdaoui, NadiaDowling, KirstenRachek, Lyudmila I.Doyle, LornaRachoń, DominikDoyle, Robert P.Rachyla, IrynaDreher, Mark L.Racine, ElizabethDrent, MarjoleinRaczkowska, EwaDrewe, JuergenRaderer, MarkusDroke, Elizabeth A.Radko, LidiaDrougard, AnneRadujkovic, AleksandarDrouin-Chartier, Jean-PhilippeRafiq, ShamailaDrożdż, WiolettaRaghu, Vikram K.Drum, ScottRagni, MaurizioDrywień, Małgorzata EwaRago, DanielaDrzymała-Czyż, SławomiraRagucci, SaraDu Preez, RyanRahbar, ElahehDu, CaiganRahi, BernaDu, HuaidongRahman, ArmanDu, ShufaRahman, SaidurDu, YixingRahman, Syed MoshfiqurDu, ZhenfangRai, NavneetDube, PrabhatchandraRai, VikrantDubowitz, TamaraRaimondi, LaviniaDuca, FrankRaimondo, DiegoDucatelle, RichardRaine, KimDucreux, SylvieRainger, George EdwardDudek, HannaRaj, DanutaDudzińska, EwaRajabi, AlaDuffy, Emily W.Rajaram, SujathaDufour, Darna L.Rajasinghe, Lichchavi DhananjayaDugar, RohitRajendran, Jagathesh ChandraDuggal, NiharikaRajendran, PraveenDumonceaux, JulieRajpurohit, SurendraDuncanson, KerithRak, AgniezkaDuncombe Lowe, KristinaRakic, JelenaDunn, CarolineRalston, Nicholas V. C.Dunn, MatthewRamachandiran, IyappanDunn, Thomas M.Ramadas, AmuthaDunneram, YashveeRamadoss, Bharathi RajaDuntas, LeonidasRamaiahgari, Sreenivasa C.Duong, Tuyen VanRamalho, Sofia M.Dupertuis, Yves MarcRamalingam, LathaDupont, JolanRamatchandirin, BalamuruganDurán, Alexandra GarcíaRamdath, DanDurazzo, AlessandraRamich (Pivovarova), OlgaDurazzo, MarilenaRamick, MeghanDurkalec-Michalski, KrzysztofRamirez, PatrickDurocher, FrancineRamirez-Graniz, Irwin AndrésDuryee, Michael J.Ramirez-Peña, EsmeraldaDusso, AdrianaRamirez-Velez, RobinsonDuszka, KalinaRamiro-Cortijo, DavidDutta, PranabanandaRamondetta, Lois M.Duttaroy, Asim K.Ramos, DomingoDuvekot, Johannes J.Ramos-Garcia, PabloDuwaerts, CarolineRamos-Morcillo, Antonio JesúsDwidar, MohammedRamos-Romero, SaraDwivedi, GarimaRampanelli, ElenaDworatzek, ElkeRamusino, Matteo CottaDwyer, Johanna T.Ranadheera, Chaminda SenakaDyakin, Victor V.Ranadheera, SenakaDymarska, MonikaRancourt, DianaDyson, PamelaRancourt, Rebecca C.Dziadek, KingaRandall-Simpson, JanisDzielska, AnnaRangan, GopiDziendzikowska, KatarzynaRanieri, MariannaE, LeziRanjan, KishuEarnest, Conrad P.Ranzato, EliaEastman, Creswell J.Rao, SheelaEaton, SimonRao, VidhyaEbadi, MaryamRapa, MattiaEbert, ScottRaposo, AntónioEck, Kaitlyn M.Rasica, LetiziaEdamakanti, ChandrakanthRasool, SuhailEdefonti, AlbertoRaszeja-Wyszomirska, JoannaEdenbrandt, Anna KristinaRatchford, StephenEdenfield, Katherine M.Rather, IrfanEdiriweera, Meran KeshawaRathi, NehaEdler, DennisRathinasabapathy, AnandharajanEdmunds, Lia R.Rathore, Atul S.Eduardo-Figueira, MariaRato, Luís Pedro FerreiraEdwards, FrancesRaval, Manmeet H.Effenberger, MariaRavanidis, StylianosEffraimidis, GrigorisRavina-Ripoll, RafaelEftekharpour, EftekharRavuri, SudheerEgan, BrendanRay, SamriddhaEgger, GarryRaybould, HelenEggersdorfer, ManfredRaz, OlgaEggler, AimeeRazzaque, Mohammed S.Eglseer, DorisRead, Scott A.Ehrlich, StefanRealdon, StefanoEid, StephanieReale, MarcellaEide, DavidReardon, Catherine A.Einarsson, SandraRebollo-Hernanz, MiguelEiros, RocioReboul, CyrilEkmekcioglu, CemRecio-Menéndez, ManuelEkström, Eva-CharlotteReed, Danielle R.Ekwaru, John PaulReed, KateEl Ghoch, MarwanReed, SpenserEl Midaoui, AdilRees, BillEl Mobadder, MarwanReginster, Jean-yvesEl Rawas, RanaRégnier, MarionElahi, FazleRegolisti, GiuseppeElam, MarcusRegula, JulitaElder, Grahame J.Rehrer, Nancy J.Eleftheriadis, TheodorosReichenbach, AlexElhadad, Mohamed A.Reichetzeder, ChristophElhai, MurielReicks, MarlaEliaz, IsaacReid, Ian R.Elimelech, EfratReigh, Nicole A.Ellen, GoversReilly, KathrynEllery, Stacey J.Rein, DietrichElli, MarinaReina Campos, MiguelEllinger, SabineReinhardt Mathiesen, ElisabethEllingsgaard, HelgaReis, FlávioElliott, BradleyRej, AnupamElliott, CharleneRejman, KrystynaElmenhorst, Eva-MariaRembiałkowska, EwaElolimy, AhmedRen, ZhenhuaEl-Remessy, Azza B.Renner, WilfriedEl-Salam, Mohamed H. AbdRenzi, SamueleEl-Salhy, MagdyRepa, AndreasEl-Say, Khalid MohamedRepsilber, DirkElsherbiny, MarwaRequicha, JoaoEl-Sohemy, AhmedRestaino, Odile FrancescaElvira-Torales, Laura InésRestrepo, BrandonEly, Brett R.Restuccia, DonatellaEmanuel, Amber S.Revuelta Iniesta, RaquelEme, PaulReynolds, ClareEmerson, Dawn M.Reynolds, Clare M.Emerson, Jillian A.Reynolds, JamesEmerson, Sam R.Reznik, SandraEmilie, T. ReasRha, Chan-SuEmmett, PaulineRhazi, LarbiEmmy Lacourt, TamaraRhee, Ki-JongEndevelt, RonitRho, JongEndre Károly, KristófRialland, MickaëlEngberink, Rik O.Riar, Amanjot KaurEngelen, Mariëlle P.K.J.Ribaldone, Davide GiuseppeEngelmann, GuidoRibaudo, GiovanniEngevik, MelindaRibeiro, ArturEngidawork, EphremRibeiro, Artur Jorge Araújo MagalhãesEnglund Ögge, LindaRibeiro, MiguelEnglund, TessaRibeiro, RogerioEngström, NiklasRibeiro, VâniaEnko, DietmarRibet, DavidEnomoto, HirayukiRiccio, GennaroEpp, LisaRiccio, PaoloErba, DanielaRiccioni, GrazianoErdman, John W.Richard, LeeEric, FinkelsteinRichards, Dylan J.Eriguchi, MasahiroRichards, GarethErnesto, Cortés-CastellRichardson, NicoleEroglu, AbdulkerimRichling, ElkeErridge, ClettRicker, EmilyErrisurriz, VanessaRicordi, CamilloErtaylan, GokhanRicotti, RobertaErwich, Jan Jaap H.M.Rideout, ToddErzurumluoglu, A. MesutRidley, EmmaEscobar, JuanRiek, AmyEscobar, Kurt A.Riemma, GaetanoEscobedo Monge, Marlene FabiolaRienecke, Renee D.Escoté, XavierRigamonti, AntonelloEser, Bekir EnginRigby, MichaelEskici, GünayRigobello, Maria PiaEskiw, Christopher H.Rigourd, VirginieEspada, MárioRiley, MalcolmEspadaler Mazo, JordiRincón, Marcos MartínEsparham, AnnaRink, LotharEsparza, M.Eugenia MartínRinninella, EmanueleEspinosa-Salinas, IsabelRios, Jaqueline LourdesEsposito, CiroRíos-Fuller, Tiffany J.Esquerra-Zwiers, AnitaRiou, JérémieEsquivel, Monica KazlauskyRishi, GautamEssen, Elisabeth VonRisi, RenataEsteban, SusanaRitz, ThomasEsteve, MontserratRitze, YvonneEstravís, MiguelRiva, AlessandraEstruch, RamónRiva, GiuseppeEstrugo-Devesa, AlbertRivera, FranciscoEsworthy, Robert StevenRivero, FernandoEufemi, MargheritaRizk, SandraEussen, SimoneRizzarelli, EnricoEva, Martínez-PinillaRizzi, LauraEvans, Christian C.Rizzo, GianlucaEvans, MalkanthiRizzolo, Diana DiazEvans, StephenRoark, Mark W.Evin, GenevièveRoato, IlariaFabbri, MarcoRobberecht, HarryFabbrocini, GabriellaRoberto, Christina A.Fabiani, RobertoRoberts, LlionFabiano, ValentinaRobertson, DeniseFabiszewska, AgataRobin, Jérôme D.Fàbregues, SergiRobino, AntoniettaFabrias, GemmaRobinson, Daniel T.Fabrizi, CinziaRobinson, EricFacchinetti, FabioRobinson, KarenFacevicova, KamilaRobinson, Stacey L.Fachim, Helene A.Robles, BrendaFagerstrøm, AsleRobles-Vera, InakiFaggian, AlessiaRobson, Shannon M.Fahey, George C.Roca Llorens, MaríaFahey, JedRocchetti, GabrieleFahrenkamp, Amy J.Rocha, Bárbara S.Fairman, CiaranRocha, Djenisa H. A.Fairweather, StephenRocha, FernandaFajkus, JiříRocha, Júlio CésarFakas, StylianosRocha-Rodrigues, SílviaFalcone, VeronicaRoche, Joseph A.Falkenburger, BjörnRock, EdmondFallacara, Dawn M.Rocks, TetyanaFalone, StefanoRockwell, MichelleFan, Hueng-ChuenRodakowska, EwaFan, JianglinRöder, StefanFan, LinlinRodilla, EnriqueFan, MeiyunRodney, RachaelFancellu, AlessandroRodrigo, LuisFang, DiRodrigues Santiago, LeandroFang, SungSoonRodrigues, Jean-MarieFanos, VassiliosRodrigues, MárcioFarabegoli, FulviaRodrigues-Mateos, AnaFarabi, Sarah S.Rodríguez Arcos, María RocíoFaraone Mennella, Maria RosariaRodriguez Lagunas, Maria JoséFaraone, ImmacolataRodriguez Sosa, Jose R.Fardet, AnthonyRodríguez Villanueva, JavierFarhat, GraceRodriguez, AdrianFarias-Pereira, RenalisonRodríguez, Anna MariaFarina, Emily K.Rodriguez, JoseFarmaki, Aliki-EleniRodriguez, LuisFarmer, NicoleRodriguez-Blanco, GiovannyFarran Codina, AndreuRodriguez-Cuenca, SergioFarrar, MarkRodríguez-García, CarmenFarrell, Stephen W.Rodríguez-Martín, Boris C.Farup, Per G.Rodriguez-Martinez, AlejandroFarzaneh, VahidRodríguez-Muñoz, Pedro ManuelFassio, FilippoRodríguez-Nogales, AlbaFasullo, MichaelRodríguez-Pérez, CeliaFattizzo, BrunoRoe, BrianFaulkner, SteveRoffman, Joshua L.Faustino, AnaRog, JoannaFava, CristianoRogers, Lynette K.Favara, GiulianaRoggero, PaolaFaviana, PinucciaRoggio, FedericoFayet, FlaviaRoghair, Robert D.Fazio, AlessiaRogus, StephanieFazzari, MarcoRohde, CarmenFeagins, Linda A.Rohde, Jeanett FriisFedosov, SergeyRohner, FabianFeeney, EmmaRohrmann, SabineFehér, AndrásRojas, DinaFei, XiangRojo-Martínez, GemmaFeighery, ConlethRoke, KaitlinFeillet-Coudray, ChristineRolf, KatarzynaFelder, Robin A.Rolfo, AlessandroFelice, FrancescaRolla, GiovanniFeng, JingRomani, AndreaFenlason, Lindy ChristineRomano, Antonino D.Fenton, TanisRomano, ClaudioFeraco, AlessandraRomano, LorenzoFerenci, PeterRomecín, PaolaFeresin, Rafaela G.Romer, AdrienneFerguson, BradleyRomero Martínez, Sonia J.Ferguson, DavidRomero, María Del MarFernandes Lemos Junior, Wilson JoséRomero-Gonzalez, BorjaFernandes, Rosa Cristina SimoesRomero-Saldaña, ManuelFernandes, RúbenRomiti, Giulio FrancescoFernandes, TitoRoncero-Martín, Raúl P.Fernández Barbero, GerardoRonkainen, JustiinaFernández Galilea, MartaRönö, KristiinaFernández Ochoa, ÁlvaroRonto, RimanteFernández, IgnacioRooney, MaryFernandez, Maria LuzRoos, EvaFernández-Bañares, FernandoRoose, GudrunFernandez-Campos, FranciscoRoot, MartinFernández-Díaz, Cristina M.Roper, Stephen D.Fernández-García, José CarlosRoques, ChristianFernández-Gómez, ElisabetRos, EmilioFernández-Martínez, EliaRosa, Gonçalo P.Fernández-Mateos, M. PilarRosa, NicolaFernández-Tomé, SamuelRosado, CarlosFernández-Villa, TaniaRosanoff, AndreaFernando, BinoshaRosdahl, JulliaFernando, Ilekuttige Priyan ShanuraRose Sadleir, KatherineFerramosca, AlessandraRose, AdamFerrando, ArnyRoseland, JanetFerranti, PasqualeRoselli, MariannaFerrar, JenniferRosendahl-Riise, HanneFerrara, NicolaRosenkranz, SaraFerrari, PietroRosenzweig, TovitFerraris, CinziaRosi, AliceFerraris, Ronaldo P.Rosinger, Asher Y.Ferraro, Ottavia EleonoraRosner, BernardFerraro, Pietro ManuelRospleszcz, SusanneFerreira-Pêgo, CíntiaRosqvist, FredrikFerretti, FrancescaRossi, FrancaFerri, NicolaRossi, MauroFerro, YveliseRossi, MichelaFerrone, FedericaRossi, NoreenFeteira-Santos, RodrigoRossi, PaolaFiates, Giovanna MedeirosRossi, Roberta ElisaFicco, Donatella Bianca MariaRostami-Nejad, MohammadFiddler, Joanna L.Roth, Christian L.Fiedorowicz, EwaRothenberg, ElisabetFields, DavidRothschild, JeffreyFife-Schaw, ChrisRoth-Walter, FranziskaFigueroa, Johnny D.Rothwell, Joseph A.Filannino, PasqualeRoubalová, RadkaFiliou, MichaelaRouch, AlexanderFilip, AdrianaRoulin, DidierFilip, AleksandraRoumeliotis, StefanosFilipiak-Florkiewicz, AgnieszkaRoumes, HélèneFinamore, AlbertoRousseau, Anne-FrançoiseFinch, MeghanRousseau, GuyFindlay, MerranRousseau, Marie-ClaudeFine, James BurkeRoverso, MarcoFine, PeterRovito, Michael J.Finer, SarahRowan, SheldonFink, Jodok MatthiasRowe, SamFinno, CarrieRowland, Maisie K.Finsterer, JosefRoy, BidishaFinucane, Francis M.Roy, Brian D.Fiol, MiguelRoy, DenisFiorentini, DianaRoy, Peter J.Fioretti, AlessandraRozerta, SokouFiorillo, LucaRuano, CristinaFiorina, PaoloRubio, CarmenFirestein, BonnieRubio, LuisFirinu, DavideRubio, Miguel AngelFischer, AlexanderRubio-Arias, Jacobo Á.Fischer, Alexander W.C.Rubio-Arraez, SusanaFischer, FlorianRucker, Robert B.Fischer, Nicholas G.Ruddock, AlanFiset, SylvainRuddock, HelenFisher, JamesRudkowska, IwonaFisher, JeffreyRudnicka, KarolinaFiszman, SusanaRudovich, Natalia N.Fitsiou, EleniRudzińska, MagdalenaFjaeldstad, Alexander W.Ruehl, RalphFlack, JohnRuel, Marie T.Flechtner-Mors, MarionRüfenacht, VéroniqueFledderjohann, JasmineRuiz Moreno, EmmaFleischhacker, SheilaRuiz, AnaFleischhacker, SheliaRuiz, Henry H.Fleischhaker, ChristianRuiz, Jonatan R.Flemming, SvenRuiz, MarioFletcher, JaneRuiz, MatthieuFletcher, JaniceRuiz-López, M. DoloresFlidel-Rimon, OrnaRuíz-Muñoz, MaríaFliss-Isakov, NaomiRuiz-Santana, SergioFlorens, NansRumpunen, KimmoFlores-Guerrero, Jose L.Rungratanawanich, WiramonFlores-Quijano, Maria EugeniaRuoppolo, MargheritaFloria, MarianaRupérez, Azahara IrisFlorowska, AnnaRuperto López, María Del MarFloyd, ElizabethRusciano, DarioFlück, JoëlleRusek, MartaFly, AlyceRuseski, JaneFogacci, FedericaRush, Elaine C.Foletto, MirtoRussell, AlanFolkvord, FransRussell, MarkFolta, SaraRussell, SimonFolwarski, MarcinRussell-Randall, KarenFomenky, Bridget E.Russo, BenedettaFonseca, Diogo A.Russo, FrancescoFontanals-Coll, MariaRusso, IlariaFontecha, JavierRusso, MariaFoong, Rachel E.Rust, Bret M.Forbes, LauraRuth, HarvieFord, DianneRutherfurd-Markwick, KayFord, Nikki A.Rutten, GeertForeman, Henry JayRuzik, LenaForestell, CatherineRyan, Alan S.Forester, Brooke E.Ryan, AnthonyForhead, Alison J.Ryan, Michael J.Forma, EwaRybalka, EmmaFormanowicz, DorotaRybicka, IgaFormisano, PietroRychlik, IvanFormoso, GloriaRyffel, BernhardFornaro, MicheleRytelewski, MateuszForslund, MariaRyu, EunjungForsse, Jeffrey S.Ryu, Jae-HaForsum, Elisabet A.Ryu, Jong HoonForsyth, AdrienneRzemieniec, JoannaForsyth, Christopher B.Saalbach, AnjaForte, GiuseppeSabbatinelli, JacopoForte, MaurizioSabia, MichaelForte, NicolaSablah, MawuliFortmann, IngmarSaccenti, EdoardoFossum, EvenSachadyn-Król, MonikaFouda, AbdelrahmanSack, UlrichFouda, SheroukSacramento, Joana F.Foureur, MaralynSadanaga, TsuneakiFourtounas, CostasSaeedi, PouyaFoutch, Brian K.Sáenz Navajas, María PilarFowler, Ashley L.Saenz, MiguelFox, MarkSaettone, VittorioFox, SimonSaez, CarmenFrade, FátimaSáez-Orellana, FranciscoFradet, VincentSáez-Padilla, JesúsFraga, Cesar G.Sagayama, HiroyukiFragkos, Konstantinos C.Saha, ProgyaparamitaFram, MaryahSaha, SanjibFrame, Leigh A.Saha, SanjoyFrancavilla, RuggieroSahpaz, SevserFrancesco, ScavelloSahu, Kamal KantFrancis, JimiSaín, JulianaFrancis, NidhishSainani, KristinFrancisco Javier, Molina OrtegaSaini, Ramesh KumarFrancisco, Javier Alvaro-AfonsoSaini, ShikhaFrank, Craig L.Sainsbury, AmandaFrank, Deborah A.Saint, Sarah E.Frank, JanSaisho, YoshifumiFrank, LuizaSaiti, AnnaFrank, SašaSaito, JumpeiFranko, AndrasSaito, KosukeFranza, LauraSaito, YoshiroFranzese, AdrianaSajdakowska, MartaFranzke, BernhardSak, Jarosław J.Fraser, DavidSakai, TakahiroFrassetto, Lynda A.Sakakibara, HiroyukiFrattaruolo, BrLucaSakamaki, AkiraFrazer, David M.Sakamoto, AtsushiFrazzi, RaffaeleSakamoto, RyuichiFreddi, LucaSakasai-Sakai, AkikoFrediani, JenniferSakayori, NobuyukiFreeman, EdwardSakharkar, PrashantFreemark, MichaelSaksena, SeemaFreiberger, EllenSakurai, KenichiFreidl, RaphaelaSala, Lucia LaFreire, MarceloSalaheen, SerajusFrezza, ClaudioSalahuddin, MelihaFrič, JanSalas, José BlancoFriedman, JacobSalas-Huetos, AlbertFrieri, GiuseppeSalazar, GloriaFriesen, CarolSalcedo, InmaculadaFriesen, Craig A.Salhab, HassanFrigstad, Svein OskarSalim, MalindaFrittitta, LuciaSalis, AmandaFritzen, Andreas M.Salm, Adrienne K.Frobert, OleSalman, MootazFrosini, MariaSalomé Pires, AnaFrost, PeterSalomone, FedericoFroyen, ErikSalonen, Bradley R.Frugé, AndrewSalto-Gonzalez, RafaelFrugé, Andrew D.Saluk-Bijak, JoannaFrutos-Fernández, María JoséSalvador-García, CelinaFthenakis, GeorgeSalvatore, FrancescoFu, Pin-KueiSalvatore, SilviaFu, QiangSalvi, PaoloFu, TingSalzano, GiovanniFu, XingSamala, Niharika R.Fu, XueyanSambri, AndreaFuchs, KlausSambri, VittorioFuchsmann, PascalSambuy, YulaFueyo-Díaz, RicardoSamir, ParimalFuge, RonSamoggia, AntonellaFuggle, NicholasSamotyja, UrszulaFuglestad, AnitaSampath, ChethanFujie, TomoyaSampedro-Núñez, MiguelFujihashi, KohtaroSamsoondar, JoshuaFujii, HidekiSan Vicente, Kontxi Martinez DeFujioka, KazumichiSanabria-Ríos, David JuliánFujiwara, AyaSánchez López, ElenaFujiwara, TomokoSanchez Vargas, Luis AlbertoFukasawa, HirotakaSánchez, AntonioFukatsu, KazuhikoSánchez, Edwin R.Fukuda, HirotsuguSánchez-Alcaraz, Bernardino JavierFukuda, MitsuruSánchez-González, José-MaríaFukushima, RyojiSanchez-Infantes, DavidFulginiti, SerenaSanchez-Luna, ManuelFuller, ScottSánchez-Muniz, Francisco J.Fülöp, TiborSanchez-Oliver, Antonio JesúsFumeaux, Céline Julie FischerSánchez-Pintos, PaulaFumitoshi, AsaiSanchez-Siles, Luis ManuelFunato, HiromasaSanchis, PilarFuncke, Jan-BerndSancho, Ana IsabelFüredi, AndrásSander, SaaraFürnsinn, ClemensSandhu, Dalbir S.Furse, SamuelSandini, MartaFurukawa, YoshikoSandlers, YanaFusaro, MariaSandoval Insausti, HelenaFusco, RobertaSandovici, IonelFyderek, KrzysztofSandusky, Peter OlafGabandé-Rodríguez, EnriqueSanfeliu, CoralGabbia, DanielaSangelaji, BahramGabbianelli, RositaSangiao-Alvarellos, SusanaGabel, KelseySangineto, MorisGabriel, BrendanSango, KazunoriGabriella, PasiniSanjeewa, Kalu Kapuge AsankaGabrielli, PaoloSankararaman, SenthilkumarGacek, MariaSankavaram, KavithaGacesa, RankoSanoudou, DespinaGaddam, Ravinder ReddyŠánová, PetraGaffney, KimSantafé, ManelGagliardi, Paolo ArmandoSantamaria, RitaGahagan, SheilaSantambrogio, PaoloGahche, JaimeSantaoja, MinnaGaillard, DanySantarpia, LidiaGaillard, RomySantarpino, GiuseppeGaitán, Adriana V.Santi-Cano, María JoséGaiz, AlmottesembellahSantorelli, Filippo M.Gajewska, AlinaSantoro, AnnaGajewska, DanutaSantoro, DomenicoGajewska, JoannaSantoro, NicolaGalan, Ana-MariaSantos López, Jorge ArturoGalanis, AlexisSantos, Carla AdrianaGalante, EvaSantos, Débora Martins DosGalassi, AndreaSantos, PauloGalelli, LucaSantos, RoseaneGaletti, ValeriaSantos-Beneit, GloriaGaliniak, SabinaSantos-Juanes, JorgeGallagher, John ChristopherSantulli, GaetanoGalli, ViolaSanz, AlejandroGallieni, MaurizioSanz-Paris, AlejandroGallo, GaetanoSaotome, MasaoGallo, LindaSaponaro, ChiaraGallo, SinaSapone, AnnaGallo-Salazar, CésarSaraiva Valente, Ana MargaridaGalmés, SebastiàSarasija, ShaarikaGamarra-Morales, YeniferSaraswathy, LakshmiyGamberi, TaniaSaraux, AlainGambo Boukary, AboubakrSaravanan, Prathab BalajiGamez, CristinaSardão, VilmaGammella, ElenaSarecka-Hujar, BeataGamon, Luke FrancisSarkar, AmritaGanann, RebeccaSarkar, AnujitGanapathy, VengateshSarkar, DipayanGangur, VenugopalSarrafzadegan, NizalGao, BeiSarria, BeatrizGao, JieSarris, JeromeGao, XiangSartini, MarinaGao, XuSartor, FrancescoGaragiola, UmbertoSartor, R. BalfourGarbowska, MonikaSartorius, TinaGarcés, CarmenSarubbo, Maria FiorellaGarcia Alonso, Maria AlejandraSasaki, GeoffreyGarcia, AdaSasaki, TakeshiGarcia, Lyda G.Sasaki, TsutomuGarcia-Bailo, BibianaSase, AjinkyaGarcía-Conesa, María-TeresaŠatalić, ZvonimirGarcia-de-la-Hera, ManoliSato, AkinoriGarcía-Galbis, Manuel ReigSato, NorikoGarcía-González, ÁngelaSato, YasutoGarcía-González, VíctorSaturnino, CarmelaGarcía-Muñoz Rodrigo, FerminSauder, KatherineGarcia-Quintanilla, AlbertSauer, Pieter JjGarcía-Ródenas, ClaraSauers, Emily J.García-Santos, José AntonioSaul, DominikGarcía-Segovia, PurificaciónSaulais, LaureGarcía-Unanue, JorgeSaules, Karen K.García-Villanova, BelénSaulnier, RonGard, PaulSaunders, BryanGarden, FrancesSauvet, FabienGardener, SamanthaSavaiano, DennisGarden-Robinson, JulieSavastano, SilviaGardner, ChristopherSavelkoul, Huub F.J.Gardner, Kevin H.Savic, DraganaGardner, ReneeSavino, FrancescoGareb, BahezSavva, George M.Gareri, PietroSawada, AkinariGaretto, StefanoSawada, ShojiroGarg, KawishSawicka, BarbaraGargari, GiorgioSawynok, JanaGarnacho Castaño, Manuel V.Saxami, GeorgiaGarner, Christine D.Sayed, Ahmed MGarofalo, NancySayer, RichardGarrido-Fernández, AntónioSayyari, AminGarrote-Adrados, José AntonioScala, IrisGartaula, GhanendraScalfi, LucaGärtner, RolandScampicchio, MatteoGarvie-Lok, SandraScandurra, RobertoGarwolińska, DorotaScannell, Amalia G.M.Garza, Jose M.Scarano, AureliaGąsecka, AleksandraScarcia, PasqualeGaspar, Renato SimõesScarpellini, EmidioGasser, OlivierScassellati, CatiaGathercole, LauraScazzina, FrancescaGatselis, NikolaosSchaafsma, AnneGaucher, CarolineSchade, HannahGaudier-Diaz, Monica M.Schaefer, MatthiasGauer, JuliaSchaftingen, Emile VanGaul, CharlySchag, KathrinGaul, DavidSchalkwijk, CasperGautheron, JérémieSchaumberg, KatherineGava, GiuliaSchauss, AlexanderGavaldà-Navarro, AleixScheck, AdrienneGawęcki, MaciejSchenk, JeannetteGaweł-Bęben, KatarzynaSchenk, PatrickGawliński, DawidScherbakov, NadjaGawron-Skarbek, AnnaScherf, Katharina A.Gazdecki, MichałScherma, MariaGazouli, MariaSchiattarella, AntonioGazzin, SilviaSchiavo, GessicaGC, ArunSchiavo, LuigiGe, WenzhenSchiefermeier-Mach, NataliaGe, XiaodongSchindler, AntonioGea Sanchez, AlfredoSchipke, JuliaGearhardt, AshleySchippa, SerenaGębski, JerzySchirmer, BastianGęca, TomaszSchlein, ChristianGeetha, ThangiahSchliemann, DesireeGęgotek, AgnieszkaSchlörmann, WiebkeGeisler, CorinnaSchloss, JanetGelzo, MonicaSchmidt, MarkGemming, LukeSchmidt, MartinGendaszewska-Darmach, EdytaSchmoll, DieterGenoni, GiuliaSchnackenberg, LauraGenovese, Kenneth J.Schneider, Stéphane MichelGentile, AmbraSchnettler, BertaGeorgakouli, KalliopiSchoeller, Dale E.George, AndersonSchoenberg, MarkusGepner, YftachScholz, ArminGeraghty, PatrickScholz-Kreisel, PeterGerards, SanneSchott, UlfGerber, Leonard E.Schöttker, BenGerber, MarietteSchrader, JörgGerding, HeinrichSchreiner, Anna J.Gerkowicz, AgnieszkaSchrezenmeir, JuergenGermano, PatriziaSchröder, AgnesGerreth, KarolinaSchröder, HelmutGervasi, MarcoSchroeder, KristaGesesew, Hailay AbrhaSchuchardt, JanGeuns, Jan M.C.Schulz, Peter J.Geyer, MartinSchulze, FriederikeGhag, GauravSchumacher, FabianGharehyakheh, AminSchumacher, Tracy L.Ghattamaneni, Naga Koteswara RaoSchuren, FrankGhemrawi, RoseSchürmann, AnnetteGherasim, CarmenSchüssler, SandraGhidini, MicheleSchwarcz, RobertGhimire, RamjeeSchwartz, GregoryGhinet, AlinaSchwartz, KennethGhiselli, AndreaSchwarzer, RalfGhosh, RajeshwarySchwerdtle, TanjaGhosh, ShobhaSciarrillo, Christina M.Ghosh, SoumitaSciascia, QuentinGiacomelli, ChiaraScicali, RobertoGiacomello, EmilianaScicchitano, PietroGiallauria, FrancescoScioli, Maria GiovannaGiammanco, AntoninaScisciola, LuciaGiammanco, MarcoScoditti, EgeriaGianesello, LisaScoppola, AlessandroGianfredi, VincenzaScorrano, PaolaGiannaccare, GiuseppeScotece, MorenaGiannelli, GianluigiScott, Fraser W.Giannini, CosimoScott, Sam N.Gianotti, LucaScott, StephanieGibas, Kelly J.Sculley, Dean V.Gibbons, JeffSdona, EmmanouelaGibby, CherylSeale, Lucia A.Gibert-Ramos, AlbertSears, BarryGibson, AliceSears, DorothyGibson, Ann L.Sebastiani, GiorgiaGibson, Deanna L.Sebastião, EmersonGibson, RachelSecades, JulioGibson, SimoneSecher, ThomasGies, IngeSechi, GianPietroGiezenaar, CarolineŠeda, OndřejGigic, BiljanaSeeds, Michael C.Giguère, YvesSeetharaman, ShyamGil, GregorioSeferidi, ParaskeviGilad, GadSegal, GadGilbert, RodneySegal, JonathanGil-Cosano, Jose J.Segarra, Ana BelénGiles, CoreySegatto, MarcoGill, HarsharnSehra, Shiv TejGill, MargaretSeidel, MariaGill, TiffanySeino, YutakaGillebaart, MarleenSeitz, Helmut K.Gillevet, PatrickSeivwright, Ami N.Gilmer, JohnSekiguchi, YasukiGiménez, María JoséSekikawa, AkiraGiménez-Gómez, PabloSekulic, DamirGioia, ChiaraSeldeen, Kenneth L.Giontella, AliceSellayah, DyanGiorgi, Alfredo DeSelvaraju, VaithinathanGioulbasanis, IoannisSempos, Christopher T.Giovannelli, PiaSemrau, KatherineGioxari, AristeaSenchina, David S.Girault, AlbanSengupta, BidishaGiri, SibSenoner, ThomasGiribaldi, MarziaSeo, Eun JiGiron-Gonzalez, Maria D.Seoane-Collazo, PatriciaGirousse, AmandineSepuru, Krishna MohanGiudetti, AnnaSequeira, Ivana R.Giudici, Kelly VirecoulonSerban, CostelaGiuliani, AngelicaSergeant, SusanGiunti, ElisaSergi, DomenicoGiusti, Anna MariaŠerić, MajaGivens, IanSerna, EvaGjerde, JenniferSerrano, Jose C. E.Gjorgjieva, MonikaSerreli, GabrieleGlabska, DominikaServais, SophieGlahn, RayServais, StephaneGlahn, Raymond P.Sessa, FrancescoGlasofer, DeborahSestak, KarolGlass Lewis, MarquisetteSestili, PieroGlauben, ThomasSevilimedu Veeravalli, SathyanarayananGlauert, Howard P.Sevil-Serrano, JavierGletsu Miller, NanaSeyfried, Thomas N.Glibowski, PawełShabnam, MominGliozzi, MicaelaShackelford, Rodney E.Glišić, BiljanaShafiee, MojtabaGlorieux, GrietShah, Birju A.Gnagnarella, PatriziaShah, Rachana D.Gnani, DanielaShah, RuchiGnessi, LucioShahar, DanitGnudi, SaverioShaheen, Abdel AzizGo, YounghoonShahi, Shailesh K.Goblirsch, Michael J.Shaikh, SibhghatullaGoda, KeisukeShaish, AvivGodbout, RogerShakerley, NicoleGoderska, KamilaShalayel, IbrahimGodos, JustynaShamieh, SaidGoeckenjan, MarenShams-White, MarissaGoeser, FelixShan, LingpengGoff, Wilfried LeShan, Zack Y.Goglia, FernandoShander, Arheh S.Golden, ChristopherShang, NanGoldman, Armond S.Shang, QingsenGoldsmith, ChloeShangguan, SiyiGoldstein, Andrew S.Shank, Lisa M.Gollub, ElizabethShankar, PadminiGolmohamadi, AmirShankland, RebeccaGolos, Thaddeus G.Shapira, NivaGomaraschi, MonicaSharma, Bhesh RajGomes, Ana CordeiroSharma, GarimaGomes, Daniela LopesSharma, GauravGomez De Agüero, MercedesSharma, GayatriGómez, Juan Carlos CastroSharma, KavitaGómez-Guzmán, ManuelSharma, NeeruGomez-Perez, Sandra L.Sharma, ShwetaGómez-Urquiza, Jose L.Sharma, VishvaGomollon, FernandoSharma, YogeshGompelman, MichelleSharp, David E.Gonçalves Ribeiro, BeatrizSharp, Matthew H.Gonçalves, CarlaSharp, PaulGondalia, Shakuntla V.Sharry, John MacGong, YunyunShashkova, ElenaGoni, LeticiaShastri, AnuradhaGonzales, Mitzi M.Shattuck, Eric ChristopherGonzález Jiménez, EmilioShaw, OdetteGonzález San José, Maria LuisaShearrer, Grace E.González Valero, GabrielShechter, AriGonzález, Encina M.Shechter, MichaelGonzález, SoniaSheen, FlorenceGonzalez-Bulnes, AntonioShehzad, AamirGonzález-Díaz, CristinaSheikh, ZubedaGonzález-Domínguez, RaúlShelley, MackGonzález-Henríquez, Juan JoséShen, Chwan-Li (Leslie)Gonzalez-Menendez, PedroShen, Szu-ChuanGonzález-Minero, Francisco JoséShen, WanGonzález-Navarro, HerminiaShen, Wen-JunGonzalez-Ochoa, GuadalupeShen, YangGonzález-Palacios, SandraShendell, Derek G.González-Soltero, RocíoSheng, JenniferGonzalez-Stuart, Armando EnriqueShepard, Blythe D.Goodla, LavanyaShepherd, SamGoodman, Richard E.Shepon, AlonGoodson, MichaelSherafat-Kazemzadeh, RoyaGoossens, YanneSherlock, Laurie G.Gor, BeverlyShertzer, Howard G.Gordon, BarbaraSherwin, Lee Anne B.Gordon, Eliza L.Shetty, AnandGordon, Paul M.Shewale, SwapnilGordon, ScottShi, HaifeiGorjanović, StanislavaShi, JiajunGorman, ShelleyShi, ZuminGórnicka, MagdalenaShibagaki, YugoGornowicz-Porowska, JustynaShibanuma, AkiraGórska-Warsewicz, HannaShibata, ShigenobuGortan Cappellari, GianlucaShibata, TomoyukiGosselet, FabienShibeeb, SaphaGostyńska, AleksandraShidoji, YoshihiroGoswami, AnandShiels, PaulGoto, KatsumasaShienfeld, YehudaGoto, KenichiShiga, YukihiroGou, JiangtaoShih, Chia-HaoGough, LewisShih, Chun-KuangGoussia, Anna C.Shim, Jae EunGouveia, Alexandra M.Shimada, MasakoGovers, CoenShimada, ShingoGowda, Siddabasave B.Shimada, YasuhitoGowey, MarissaShimizu, MakotoGoya, LuisShimizu, TakahikoGoytia, MairaShimizu, YujiGozal, EvelyneShimomura, YoshiharuGrabacka, MajaShimpi, NeelGrabauskas, GintautasShimshoni, YaaraGrabek-Lejko, DorotaShin, AndrewGrabrucker, AndreasShin, DongmiGraça, Flávia AparecidaShin, Myung-junGrace, FergalShin, SangahGrace-Farfaglia, PatriciaShinde, TanviGracia, IgnacioShinsugi, ChisaGraef, JenniferShiraishi, Jun-ichiGrafenauer, SaraShirin, SoniaGraham, AndreaShive, SteveGrajower, Martin M.Shkodra, MorenaGramer, GwendolynShlisky, Julie D.Gramlich, LeahShmarakov, IgorGrammatikopoulou, Maria G.Shoaie, SaeedGranado-Casas, MinervaShoben, Abigail B.Granados-Principal, SergioShoji, HiromichiGranata, RicardaShokal, UpasanaGrandinetti, AndrewShokouh, PedramGranero, RoserShomura, MasakoGrangette, CorinneShukitt-Hale, BarbaraGranholm-Bentley, LottaShukla, AlpanaGranic, AntonetaSi, HongweiGranica, SebastianSiafarikas, ArisGrant, Maria B.Sibhatu, Kibrom TadesseGrant, RossSicińska, EwaGrant, WilliamSiddall, AndyGrant, William BurgessSiddique, Abu BakarGrasa, LauraSieber, FritzGrases, FelixSiegel, Jessica A.Grassi, IlariaSiegel, Robert M.Grąt, KarolinaSiener, RoswithaGrattagliano, IgnazioSierra-Campos, ErickGraudal, Niels AlbertSikorska, EwaGray, Heewon LeeSikorska-Zimny, Kalina MajaGray, NicolaSilaghi-Dumitrescu, RaduGray, SelenaSilano, MarcoGraziani, VittoriaSilva, Ana FilipaGrech, AmandaSilva, LuísGreco, LuigiSilvagno, FrancescaGreen, BenjaminSilvestre, Marta Filipa PaulinoGreen, DonSilvestre, Marta P.Green, HilarySilvestri, CristoforoGreen, MarkSimenko, JozefGreenberg, DanielleSimeone, PaolaGreene, Michael W.Simes, DinaGreenough, AnneSimiczyjew, AleksandraGreer, BeauSimões-Wüst, Ana PaulaGreer, Frank R.Simón Magro, EdurneGregori, DarioSimon, EdurneGregory, Richard L.Simon, JorgeGrenda, TomaszSimon, Marie ChristineGressier, Mathilde M.Simoni, JanGrgic, JozoSimón-López, Leticia CarmenGriauzde, Dina HafezSimons, Sinno HPGrider, ArthurSimonsen, Anja HviidGridneva, ZoyaSimper, TrevorGrieger, Jessica A.Simpson, LizGriffin, LauraSimpson, Melanie RaeGriffin, NicholasSimpson, SteveGriffin, Nicholas W.Sims, BrianGriffith, Brian NelsonSims, Clark R.Grigorescu, Elena-DanielaSims, StacyGrim, Clarence E.Sims-Robinson, CatrinaGrimaccia, ElenaSina, ChristianGrimm, AmandineSince, MarcGrimm, MarcusSinclair, AndrewGriñán-Ferré, ChristianSinclair, DuncanGrippo, Paul J.Sinclair, MarieGrochowski, CezarySinger, PierreGrody, Wayne W.Singh, AnilGroer, MaureenSingh, ArashdeepGromadzka-Ostrowska, JoannaSingh, BanoGrošelj, UrhSingh, ChandraGross, Susan MichelleSingh, PratibhaGrosse, Gerrit M.Singh, RajbirGrossini, ElenaSingh, Santosh KumarGrossmann, RuthSinghal, AtulGrosso, GiuseppeSingla, BhupeshGroufh-Jacobsen, SynneSinharoy, PritamGruber, Kenneth J.Sinko, PatrickGruber–Bzura, Beata M.Siobhan Mitchell, EllenGrubić Kezele, TanjaSioofy-Khojine, Amir-BabakGruère, GuillaumeSiopis, GeorgeGrundy, MyriamSiotto, MariacristinaGrygier, AnnaSipos, KatalinGrzelak, TeresaSiracusa, RosalbaGrzybowski, AndrzejSiracusano, SalvatoreGrzywacz, AgataŠirić, IvanGualdi-Russo, EmanuelaSiriwardhana, NalinGuan, QingdongSisti, DavideGuan, VivienneSivalingam, PeriyasamyGuandalini, StefanoSivaprakasam, SathishGuarino, MariaSiziba, LindaGuarino, Michele Pier LucaSjödin, Anders MikaelGubatan, JohnSjoholm, AkeGubbi, SriramSkelly, DeanneGude, FranciscoSkelton, KaraGuéant, Jean LouisSkenderi, Katerina P.Guenther, PatriciaSkinner, ChristopherGuerreiro, Catarina SousaSkoczylas, ŁukaszGuerrieri, FrancescaSkoglund, KristoferGuers, John J.Skonieczna-Żydecka, KarolinaGuertler, AnneSkouroliakou, MariaGueven, NuriSkowrońska, KatarzynaGuglielmo, PriscillaSkröder, HelenaGugliucci, AlejandroSkrzep-Poloczek, BronisławaGuha, PrasunSkypala, IsabelGuillier, LaurentSlavin, Joanne L.Guilloux, Jean-PhilippeSledzinski, TomaszGuligowska, AgnieszkaSleet, DavidGulino, Ferdinando AntonioSleigh, JamesGulliver, AmeliaSlim, KaremGunaje, JayaramaSlim, SmaouiGunaratne, NadeeshaSlimings, ClaudiaGunasekar, Susheel K.Śliwerski, AndrzejGundamaraju, RohitSliwinski, TomaszGundert-Remy, UrsulaSlominski, AndrzejGunst, JanSlowing, KarlaGunter, Jennifer H.Słupecka, MonikaGunton, JennySlyvka, YuriyGuo, Chih-HungSmalla, Karl-HeinzGuo, Jong-LongSmethers, AlissaGuo, PenghongSmeuninx, BenoitGuo, WeiminSmiley, AbbasGuo, YanhongSmith, Albert F.Guppy, FergusSmith, BarryGupta, AdyyaSmith, BrendaGupta, AnshuSmith, Brittany L.Gupta, NehalSmith, Bryan K.Gupta, NilakshSmith, ClaireGupta, RaviSmith, GordonGupta, SanjaySmith, JeffreyGurdeniz, GozdeSmith, Jessica D.Guriec, NathalieSmith, Kathryn E.Gussoni, MaristellaSmith, LoisGustafson, Christopher R.Smith, Stephen B.Gustafsson, JennySmith, Webb AGustafsson, PeikSmits, TimGuthrie, JoanneSmoak, PeterGutíerrez Hervas, Ana IsabelSmoluk-Sikorska, JoannaGutiérrez-Juárez, RogerSmyth, PeterGutkowska, KrystynaSneddon, JenniferGuzek, DominikaSnelson, MatthewGuzmán Ruiz, RocíoSnow, DeniseGuzzi, FrancescoSnyder, RuthGwin, Jess A.So, Po-WahGwynne, KylieSoares, RogérioGyamfi, Maxwell A.Sobeh, MansourH. Shirvani, ArashSobieraj, PiotrHa, Jung-HeunSobocanec, CSandraHa, KyunghoSobota, AldonaHa, Min-SeongSobue, TomotakaHaaparanta-Solin, MerjaSocha, KatarzynaHaas, VerenaSogawa, NorioHaase, HajoSokal-Gutierrez, KarenHabek, MarioSokolowska, MilenaHaboubi, NadimSolanki, SumeetHabte-Tsion, Habte-MichaelSolano-Aguilar, GloriaHachisu, YoshimasaSolesio Torregrosa, Maria De La EncarnacionHackett, DanielSoleti, RaffaellaHackman, LavenderSolomon, NancyHage, RenéSolon-Biet, SamanthaHagedorn, Rebecca L.Somoza, BeatrizHagell, AnnSong, In GyuHaghiri, MortezaSong, In-KyungHagihara, KeisukeSong, JiajiaHagner-Derengowska, MagdalenaSong, Jong KookHahn, AndreasSong, Ping (Cleveland Clinic Foundation, USA)Hahn, Robert G.Song, Ping (Georgia State University, USA)Hailer, KatieSong, RuiHaines, JessSong, TongxingHains, DavidSong, YoonJuHair, Amy BoriskieSoni, Kiran KumarHakkak, RezaSoreq, HermonaHakovirta, Harri H.Soriano Maldonado, AlbertoHałasa, MaciejSoriano, José MiguelHaldar, SumantoSoriano, SalvadorHaldeman, LaurenSorkin, BarbaraHalfwerk, SusanneSorrenti, VincenzoHall, DuaneSosa, EricaHall, SusanSotek, ZofiaHallmann, EwelinaSoto, Esther CuadradoHalmos, Emma P.Sotomayor, Camilo G.Ham, DanielSouček, PavelHamano, TadanoriSourij, HaraldHamaoka, TakafumiSousa Lima, InêsHameed, AhsanSousa Martin, CarolinaHamlin, RobertSousa, MónicaHampel, DanielaSouza, Felipe Da CostaHamułka, JadwigaSowa, IreneuszHan, AlexSowndhararajan, KandhasamyHan, SeungYongSozańska, BarbaraHan, YoungjiSpaeth, AndreaHanas, JaySpagnuolo, CarmelaHanf, JonSpagnuolo, RoccoHanganu, DanielaSpallotta, FrancescoHanisch, Franz-GeorgSpanier, BrittaHankir, Mohammed K.Spano, GiuseppeHanley-Cook, GilesSpartalis, EleftheriosHanlon, Erin C.Spartalis, MichaelHanna, RamySpath, Hans-MartinHannibal, LucianaSpeck, MelanieHans, GuySpencer, MelanieHanschen, FranziskaSpencer, NeilHansen, Anita L.Spera, GiovanniHansen, DitteSperanza, EnzaHansen, Keith A.Sperber, G. H.Hanson, JenniferSperotto, FrancescaHanson, LisaSpiegler, JulianeHansrivijit, PanupongSpiller, RobinHao, JiaqingSpinnato, PaoloHaqq, AndreaSpisni, EnzoHara, AkinoriSpiteri-Cornish, LaraHarasym, JoannaSpitznagel, Mary BethHardaway, AndrewSplichal, IgorHarding, CeliaSprajcer, MadelineHarding, ScottSpriet, LawrenceHardman, Roy J.Squillacioti, GiuliaHardt, JessicaSquitti, RosannaHardwick, James P.Sremanakova, JanaHareendran, SangeethaSridhar, SubbaramiahHargreaves, IainSrinivasagan, RamkumarHaring, BernhardSrinivasan, BharaniHarmanci, Arif OzgunSrinivasan, VijayHarmon, Brook E.Srivastava, SaurabhHaron, MonaSriwastva, Mukesh KumarHarper, GordonSroka, ZbigniewHarris, CarlaSt John, James A.Harris, Edward N.Staal, JensHarris, Erin P.Stabouli, Stella V.Harris, NeilStachowska, EwaHarris, William S.Stadlbauer-Köllner, VanessaHarrison, Fiona E.Stadnyk, AndrewHart , Prue H.Staege, Martin S.Hart, KathrynStaiano, AnnamariaHärtel, ChristophStanforth, Philip R.Hartmann, James X.Stange, KatjaHartwell, Jennifer L.Stangl, GabrieleHarty, PatrickStangl, HerbertHaruhara, KotaroStaniek, HalinaHaruna, MegumiStanirowski, Paweł JanHarvey, LouiseStanislawska-Sachadyn, AnnaHarvie, MichelleStanstrup, JanHasan, ProttoyStanton, RichardHasegawa, YokoStanton, RosemaryHasegawa, YuhStappen, IrisHashimoto, KenjiŠtarha, PavelHashimoto, KoichiStark, AlizaHashimoto, NaotoStark, Felicity C.Hashimoto, TakeshiStarościak, WojciechHashimoto, YoshitakaStasiak-Różańska, LidiaHaskell-Ramsay, CrystalStaszewski, OriHaslam, DanielleStaub, DanielHaß, UlrikeStaunstrup, Nicklas HeineHassan, Amr AbdulfattahStauss, Harald M.Hassan, IbrahimStavinoha, Peter L.Hasselblatt, PeterStavrinou, PinelopiHata, AkihisaStay, SharonHatch, ElizabethStec, David E.Hatta, HideoŠtefan, LovroHattori, YuichiStefan, NorbertHaub, MarkStefanidou, Maria E.Hausien, AmrStefanska, AnnaHausken, TrygveSteffen, JuliusHauta-alus, Helena H.Stefler, DenesHawa, RaedStehle, PeterHay, IanStein, ChristophHayasaka, KiyoshiSteinberg, FranceneHayashi, KaoriSteinhoff, UlrichHayashi, MikioStengel, AndreasHayashi, ShyunjiŠtěpánek, LadislavHayes, KCStephens, Jacqueline M.Haymann, Jean-PhilippeStepien, Karolina M.Haynes, JenniferStergios, KonstantinosHays, NicholasSteri, MaristellaHayward, JoshuaStetina, Birgit U.He, FangStevens, DavidHe, FengjunStevens, RebekahHe, Jia-QiangStevenson, LeoHe, LixiaStewart, LauraHe, MingyuStiuso, PaolaHe, XiaochenStoecker, BarbaraHealey, GenelleStojek, MonikaHearst, MaryStoller, Marshall L.Heath, AliciaStone, Trevor WilliamHeberden, ChristineStookey, JodiHebert, JamesStornaiuolo, MarianoHeckmann, JosefStørsrud, StineHeckmann, MatthiasStote, KimHegazi, RefaatStothard, Ellen R.Hegyi, Thomas HegyiStovall, Daniel B.Heijboer, Annemieke C.Støving, René KlinkbyHeil, Sandra G.St-Pierre, David H.Heinen, MirjamStragierowicz, JoannaHeiss, CindyStrandberg, TimoHeißel, AndreasStrandvik, BirgittaHekmatdoost, AzitaStraniero, SaraHelena Siegel, MarciaStrappe, PadraigHeller, GerwinStrasser, BarbaraHellmann, Jason L.Strati, FrancescoHellstrom, PerStrazzullo, PasqualeHemachandra, Reddy P.Streker, MeikeHemilae, HarriStriano, PasqualeHemmingway, AndreaStringham, James M.Hendifar, Andrew E.Strommer, SofiaHendrich, SuzanneStruga, MartaHenes, Sarah T.Struijk, Ellen AmandaHennigar, StephenStrunecka, AnnaHenrick, BethanyStubbs, James R.Henry, Ryan A.Stubert, JohannesHerda, AshleyStuetz, WolfgangHerington, Jennifer L.Stull, April J.Hermansen, KjeldSuárez-Llanos, José PabloHernáez, AlvaroSubbaiah, Papasani VenkataHernandez, AbbiSubedi, LalitaHernández, AdrianaSubramanian, ParthasarathiHernandez, ArturoSubramanian, SowmyalakshmiHernández-Alonso, PabloSubramanian, VeedamaliHernández-Breijo, BorjaSuen, Jacky Y.Hernández-Hernández, OswaldoSuez, JothamHernández-Ruiz, AngelaSugawara, AkihiroHerraez, ElisaSu-Hsin, ChangHerrmann, MarkusSukocheva, OlgaHerscovitz, HayaSukumar, DeepthaHershberger, KathleenSukumar, PiruthiviHess, Julia MeredithSukumaran, Sunil KumarHettihewa, Sujeewa K.Sulentic, CourtneyHew-Butler, TamaraSuliburska, Joanna MariaHewelt-Belka, WeronikaSuliga, EdytaHewlings, Susan J.Sulmont-Rosse, ClaireHeydenreich, JulianeSumiyoshi, EriHeyse, AlexSummers, Katie LynnHickey, AmandaSun, ChengwenHieronimus, BettinaSun, Feng-HuaHiesmayr, MichaelSun, GuangHigashi, TomomiSun, JiadongHigashida, KazuhikoSun, RamonHiggins Neyland, MarySun, XiaofeiHii, Charles S.Sun, YangboHilakivi-Clarke, LeenaSun, YitangHildebrand, DeanaSundaram, KruthikaHilger, JenniferSundaramoorthy, PasupathiHilhorst, MarcSundekilde, Ulrik K.Hill, AileenSung, Hoon-KiHill, Alison M.Sunny, Nishanth E.Hill, CameronSuominen, Sakari B.Hill, DavidSurapaneni, Krishna MohanHillier-Brown, Frances C.Surendran, SushithaHimmerich, HubertusSuriano, FrancescoHimoto, TakashiSuryadevara, VidyaniHingorani, RittuSuryavanshi, Santosh V.Hininger-Favier, IsabelleSutoh, YoichiHippensteel, JosephSuwalska, AleksandraHirano, KatsuyaSuzuki, HideoHirasaka, KatsuyaSuzuki, KatsuhikoHirasawa, MichiruSuzuki, MasakoHiroaki, HidekazuSuzuki, ShuichiHirsch, Katie R.Suzuki, ToshikazuHisatsune, TatsuhiroSvoboda, LaurieHissa, BarbaraSvoboda, MartinHitachi, KeisukeSwami, UmangHjorth, MaritSwaminathan, ArunHo, ChitangSwanepoel, LibbyHo, Chi-TangSwindle, Taren M.Ho, Shih-ChingSwitkowski, KarenHo, Tin-YunSwoap, StevenHo, VincentSych, JaniceHobin, ErinSyed, FarooqHoddy, KristinSyed-Abdul, Majid MufaqamHodge, AllisonSylvetsky Meni, AllisonHoerr, RobertSymonds, Michael E.Hoffman, DeniseSytykiewicz, HubertHoffman, JessicaSyurina, ElenaHoffman, RichardSzablewski, LeszekHoffmann, PeterSzamotulska, KatarzynaHoffmanová, IvaSzczepanek, JoannaHoge, AxelleSzczepanek, KingaHogenelst, KoenSzczerbinski, LukaszHogervorst, EefSzerszunowicz, IwonaHøjfeldt, GrithSzewczyk, Nathaniel J.Hojsak, IvaSzewczyk-Golec, KarolinaHolasek, Sandra JohannaSzilagyi, AndrewHolden, ShelleySzkudelski, TomaszHoleček, MilanSzkup, MałgorzataHolland, JustinSzlagatys-Sidorkiewicz, AgnieszkaHolland, Michelle L.Szostak-Węgierek, DorotaHolland-Winkler, Angelia MaleahSzulc, AgataHollar, Theodore LucasSzulc, KarolinaHollenbach, MarcusSzulińska, MonikaHollis, JamesSzychlinska, Marta AnnaHolloway, KaraSzymanski, ChristineHolmberg, ChristopherTabara, YasuharuHOLMES, ROSS P.Tabassum, RubinaHolsinger, DamainTabatabaei-Jafari, HosseinHolt, DarcyTaber, Christopher B.Holt, Marie K.Tabler, Jennifer LynHolt, RobertaTacke, FrankHolvik, KristinTae-Jin, KimHolwerda, AndrewTafani, MarcoHolzapfel, ChristinaTaft, Diana HHomko, Carol J.Tafuri, SimonaHommel, Jonathan D.Tagliabue, AnnaHonda, YasushiTaglietti, ValentinaHong, Sung NohTaguchi, KenseiHooshmand, BabakTai, Dar-FuHopkins, BrettTaibi, AmelHopkins, LauraTain, You-LinHopperton, KathrynTajiri, YujiHord, Norman G.Tak, Young JinHorgan, GrahamTakada, TappeiHoriguchi, HyogoTakado, YuheiHormes, Julia M.Takagi, KoseiHorne, Benjamin D.Takahashi, NobuyukiHornik, BeataTakahashi, YumikoHorrigan, Louise A.Takaki, AkinobuHorstmann, AnnetteTakamura, NoboruHortigón-Vinagre, MaríaTakano, KatsuraHorvath, AngelaTakata, TomoakiHoshiko, HeatherTakata, YumieHoshino, DaisukeTakatani-Nakase, TomokaHosojima, MichihiroTakaya, JunjiHosoyamada, MakotoTakazawa, ShinyaHossain, Md EkhtearTakegahara, NorikoHossain, Md JakirTakenaga, MitsukoHossain, SharminTakimoto, HidemiHossain, ZakirTako, EladHosseinpour, SepantaTalati, ZenobiaHou, RuixueTalbot, ShawnHouben, TomTallis, JasonHouchins, JennyTalsma, Elise F.Houshdaran, SaharTam, Jonathan S.Houston, Mark C.Tamayo, EstherHoward Wilsher, StephanieTamm, Anna-liisaHoward, Annie GreenTamura, YoshiakiHowarth, GordonTamura, YukinoriHowe, Caitlin G.Tan, Bee K.Howe, StephanieTan, Bee KangHowell, BrianTan, IsabellaHoy, Mary KatherineTan, LiboHronek, MiloslavTan, RachelHruz, Paul W.Tan, RuirongHsiao, Po-JenTan, Shu LingHsieh, Chia-ChienTan, Sze YenHsieh, PeilingTan, YutingHsieh, Tusty-JuanTanabe, KatsuyukiHsieh, Yi-ChenTanaka, AtsushiHsu, Bang-geeTanaka, SatoshiHsu, Chin-LinTanaka, TakujiHsu, Fang RongTanaka, ToshikoHsu, Shu-haoTanasova, MarinaHsu, Yuan-ManTande, DesireeHu, ChanglingTang, JonathanHu, GuoliTang, MinghuaHu, MinTang, XiaoleiHu, TianTang, YaoHu, WeisyunTangadanchu, Vijai Kumar ReddyHu, XiaoyuTangney, Christy C.Hu, YinanTani, YukakoHu, YingTanner, Simpson BoboHu, YuTannock, GeraldHu, YuanTanui, Collins K.Huang, Chi-ChangTanzilli, GaetanoHuang, Chien-ChungTao, ChenqiHuang, Chun-YungTao, Meng-HuaHuang, Fu-ChenTapia Serrano, Miguel ÁngelHuang, Hui-ChunTappia, ParamjitHuang, Jiun-ChiTapsoba, FrançoisHuang, Li-TungTarantini, StefanoHuang, MengnaTarantino, GiovanniHuang, Paul Li-HaoTardy, Anne-LaureHuang, Shih-YiTargońska-Stępniak, BożenaHuang, Tse-HungTarleton, Emily K.Huang, Wei-ChunTarpey, MichaelHuang, Wen-ChungTarragon Cros, ErnestoHuang, XiaohuTartera, CarmenHuang, XinTartey, SarangHuang, YanTashani, Osama A.Huang, Yi-ChenTauber, JamesHuang, Yi-ChiaTauriello, DanieleHubbard, ElizabethTavares, LucianaHuber, AndrewTawia, SusanHuber, MichaelTayek, John A.Hudson, GeoffreyTayie, FrancisHudson, Joshua L.Taylor, Briana J.Hudzik, BartoszTaylor, Carla G.Huedo-Medina, Tania B.Taylor, Harrison B.Huërou-Luron, Isabelle LeTaylor, Pennie J.Huerta-Yépez, SaraTaylor, RachaelHuffman, Kelly J.Taylor, Rachael M.Huffman, MarkTecce, Mario FeliceHughes, CatherineTedstone, AlisonHuh, Jin WonTeichert, JoannaHuh, Joo YoungTeigen, LeviHulme, JohnTeišerskas, JustinasHulsey, ThomasTeisseyre, AndrzejHulshof, ToineTeixeira, LeandroHuneau, Jean FrançoisTejpal, ShilpaHung, Shao-WenTelang, SuchetaHung, Szu-ChunTempfer, HerbertHunjadi, MonikaTemple, Jennifer L.Hunot, ClaudiaTen Have, GabriellaHunsberger, MonicaTencerova, MichaelaHunt, DavidTeng, PengHunt, JanetTeng, Ru-JengHunt, NCTeng, YongHunt, WendyTenkorang, MavisHuppé-Gourgues, FrédéricTenore, Gian CarloHur, Yang-ImTeodoro, Anderson JungerHurst, RogerTeodoro, JoãoHurwitz, Lisa B.Tepper, SigalHuston, Robert K.Terauchi, MasakazuHutchinson, KatrinaTerranegra, AnnalisaHutchison, Amy T.Teruel-Montoya, RaúlHuys, Geert R.B.Teruyuki, TanakaHuysentruyt, KoenTerzuoli, LuciaHwang, Chueh-LungTeschke, RolfHwang, Daniel Liang-DarTeske, JenniferHwang, In KooTessari, PaoloHwang, Keum TaekTessema, MasreshaHwang, Lee-ChingTessier, FredericHwang, Liang-DarTetens, IngeHyatt, HaydenTey, Siew LingHyde, NatalieThakali, KeshariHyldig, GretheThangaraj, AnnaduraiHypponen, ElinaThanikachalam, KannanHytti, MariaThavamani, AravindHyun, TaisunTheiler-Schwetz, VerenaIacone, RobertoThen, CorneliaIatsenko, IgorTheodorou, GeorgiosIbarra, AlvinThi Thu Nguyen, ThaoIbrahim, FandiThielecke, FrankIchihashi, MasamitsuThijs, CarelIchikawa, HiroshiThirunavukarasu, DeepakIckes, ScottThirunavukkarasu, ShyamalaIde, KazukiThoene, MelissaIerardi, EnzoThoma, ChristianIgarashi, MikiThomas, CharlesIglesia, IrisThomas, JenniferIglesias-Osma, Maria CarmenThomas, TiffanyIizaka, ShinjiThomas, WayneIizuka, KatsumiThompson, Campbell H.Ijiri, DaichiThompson, Henry J.Ikedo, AoiThompson, MichaelIkegaya, NaokiThompson, NickIkura, YoshihiroThongprayoon, CharatIkuta, ToshikazuThorisdottir, BirnaIlag, Leodevico L.Thornley, SimonIlaria, MarsilioThornley-Brown, DenyseIlesanmi-Oyelere, Bolaji LilianThornton, CatherineIm, Syn-HeyogThornton, Margaret JulieImai, HirooThorsby, Per MedbøeImai, ToshioThorsdottir, IngaImbard, ApollineThorwald, MaxImbimbo, BrunoThota, RohithImig, JohnThuzar, MoeImpellizzeri, DanielaThyagarajan, BaskaranImperatori, ClaudioTian, YuanInafuku, MasashiTiao, Mao-MengInchingolo, FrancescoTicconi, CarloIndrio, FlaviaTicha, AlenaInfante, MarcoTicinesi, AndreaInglis, Julia E.Tickell, Kirkby D.Ingram, DonaldTieland, MichaelIngram, Katherine H.Tieri, MariaInns, StephenTiffon, CélineInoue, HirofumiTiidus, PeterInoue, IkuoTiller, NicholasInoue, KazukiTimmerman, KyleInoue, TatsuroTinanoff, NormanInsenser, MaríaTincati, CamillaInvernizzi, MarcoTinggi, UjangIoakeimidis, IoannisTiselius, Hans-GoranIoannou, PetrosTitcomb, Tyler JIovino, PaolaTitz, BjoernIrakli, MariaTkacz, KarolinaIrawati, DeasyTkaczewska, JoannaIriondo, AmaiaTobberup, RandiIsenberg-Grzeda, ElieTobina, TakuroIseppi, LucaTobis, SlawomirI-Shiang, TzengToda, KazuyaIshibashi, KenichiTodd, JoshuaIshihara, KengoToedebusch, ChristineIshihara, ToruTokarek, TomaszIshikawa, MidoriToledo Atucha, EstefaníaIskra, MariaToledo-Corral, ClaudiaIskusnykh, Igor Y.Toma, KumikaIslam, Md SorifulTomah, ShaheenIslam, Mohammad MazharulTomaiuolo, RossellaIslam, Mohammad ZahirulTomasello, GiovanniIsmael, SaifudeenTomassoni, DanieleIsozaki, TakeoTomaszewska, EwaIspoglou, TheocharisTomczak, SzymonIssaka, Abukari ITomczykowa, MonikaIstas, GeoffreyTomkin, GeraldIstfan, NawfalTomlinson, DavidItani, LeilaTong, JessicaItariu, Bianca KarlaTong, TammyIto, FumiakiToomer, OndullaIto, NaokiToorie, AnikaIto, TomokoTooze, JanetIvanov, AndreiTopping, DavidIvanova, EkaterinaToral, MartaIversen, Per OleToribio-Mateas, MiguelIvy, JessToriyama, MichinoriIvy, John L.Toro Martín, Juan DeIwahori, ToshiyukiTorquati, LucianaIwasaki, MasanoriTorreggiani, MassimoIweala, OnyinyeTorrens-Mas, MargalidaIyer, ChitraTorres López, M. IsabelIzawa, Kazuhiro P.Torres, DuarteIzawa, TakeshiTorres, MoisesIzquierdo Álvarez, SilviaTorres, NimbeIzquierdo Pulido, MariaTorres, SandraJ. Palmer, DebraTorres, SusanJ. Pen, JoeriTorres-Collado, LauraJabłońska, BeataTørris, ChristineJablonska, EwaTosato, MatteoJaceldo-Siegl, KarenToscano, MassimilianoJackson, ChandraTota-Maharaj, RajeshJackson, PhilippaTotosy De Zepetnek, JuliaJacquemin, LaureTouil-Boukoffa, ChafiaJacques, HélèneTovoli, FrancescoJadavji, NafisaTownsend, StevenJadavji, Nafisa M.Toyama, IkuoJadhav, ShyamalagauriToyama, TadashiJaeger, RalfToyoda, MasashiJaehne, Anja KathrinToyoda, YuJafari, JafarTozzoli, RenatoJagga, SupriyaTraber, MaretJahandideh, ForoughTrabold, NicoleJahan-Mihan, AlirezaTrabulsi, JillianJain, AjayTraczyk, IwonaJain, AnkurTraglia, MichelaJain, AnshikaTraina, GiovannaJakaria, Md.Trakman, GinaJakobsen, MogensTrammell, SamuelJakubczyk, KarolinaTran, PhuJalil, Abbe Maleyki MhdTranchant, CaroleJalili, ThunderTrani, AntonioJalili-Moghaddam, ShabnamTrapani, ValentinaJamar, GiovanaTrautwein, ElkeJames, Gary D.Travers, Jeffrey BryantJames, JoelTraylor, Daniel A.James, LoisTrehan, IndiJameson, EleanorTrelis, MaríaJamieson, Emma L.Tresguerres, JesúsJamieson, Jennifer A.Trewin, AdamJamka, MałgorzataTriantafillidis, John K.Jamshed, HumairaTriantafyllidis, AndreasJang, CholsoonTriantafyllou, KonstantinosJanikowska, GrażynaTrico, DomenicoJaniszewska, MariolaTrif, MonicaJankauskiené, AugustinaTriggiani, VincenzoJankovic, NicoleTrijsburg, LauraJankovic-Karasoulos, TanjaTrinchese, GiovannaJankowska, MagdalenaTrindade, EuniceJannasch, FranziskaTripathi, Lokesh P.János, RigóTripathi, Siddharth KaushalJanovska, PetraTripicchio, GinaJansen, Erica C.Tripolt, NorbertJansen, Lisa T.Triunfo, StefaniaJanssen, RobTrivli, AlexandraJanus, EdwardTroen, AronJanzen, NilsTroesch, BarbaraJaradat, NidalTroisi, JacopoJarak, IvanaTrommelen, JornJarbrink-Sehgal, Maria EllionoreTroy, LisaJarocka-Cyrta, ElzbietaTruby, HelenJarzebski, MaciejTrufero, Javier MartínezJasdanwala, SarfarazTruman, EmilyJaszek, MagdalenaTruskey, George A.Javaheri, TaherehTrybek, PaulinaJaworowska, AgnieszkaTrząskowska, MonikaJay, Jennifer A.Tsabouri, SophiaJayatissa, RenukaTsai, Chang-YouhJe, Jae-YoungTsai, Li-TangJeckel, Kimberly M.Tsai, Meng-CheJedrejek, DariuszTsai, ShihfengJegatheesan, PrasanthiTsai, Ying-ChiehJekiełek, MałgorzataTsakiridis, IoannisJelińska, AnnaTsaniklidis, GeorgiosJelski, WojciechTsaousidou, EvaJelsma, JudithTsapanou, AngelikiJen, Kai-Lin CatherineTsay, Shwu-ChenJena, Prasant KumarTsiani, Evangelia LitsaJenkins, DavidTsien, CynthiaJensen, BenjaminTsikouras, PanagiotisJenzer, HelenaTsirka, Styliani-Anna (Stella)Jeon, SookyoungTsivgoulis, GeorgiosJeong, DongtakTsochatzis, Emmanouil D.Jeong, Seon-YongTsofliou, FotiniJeppesen, Per BendixTsolakis, Apostolos V.Jernerén, FredrikTsopmo, ApollinaireJeruszka-Bielak, MartaTsubota, AkihitoJerzy, MosiewiczTsubota, KazuoJeschke, UdoTsuda, HiroyukiJezela-Stanek, AleksandraTsuduki, TsuyoshiJeżewska-Zychowicz, MarzenaTsugawa, HitoshiJi, XiangmingTsuji, YousukeJi, YuelongTsukahara, TakamitsuJian, XingTsukamoto, HayatoJiang, PeihuaTsuyoshi, TsudukiJiang, RulanTu, Hung-PinJiang, XinyinTucher, EmmaJiang, XiqianTucker, JaredJiannine, LiaTucker, Robin M.Jiao, LiTueros, ItziarJimbow, KowichiTufarelli, VincenzoJimenez Del Val, IoscaniTúnez, IsaacJimenez-Chillaron, JosepTung, Tao-HsinJiménez-Moleón, José J.Tuomilehto, JaakkoJiménez-Morales, MònikaTurdo, AliceJiménez-Ortega, VanesaTurk, MelanieJimoh, FlorenceTurner, JonathanJimoh, Oluseyi F.Turner, JustineJin, Hyun-SeokTurner, LindseyJin, LiTurner, Raymond ScottJin, MirimTurner, Russell T.Jing, RanTurner-McGrievy, Gabrielle M.Jodynis-Liebert, JadwigaTurni, ConnyJoe, MillwardTurpin, WilliamsJohansson, IngegerdTurpin-Nolan, SarahJohnson, Brittany J.Turrini, AidaJohnson, Elizabeth J.Turski, WaldemarJohnson, IanTveden-Nyborg, PernilleJohnson, Ian T.Tvedt, Tor Henrik AndersonJohnson, LauraTye-Din, JasonJohnson, RichardTzanakis, Ioannis P.Johnson, Sarah A.Tzen, Jason T.Johnson, Tricia J.Tzeng, Huey-MingJohnson-Mann, CrystalTzeng, Shiang-JongJohnston, CarolTzvetkova, PavletaJohnston, EmilyUberti, DanielaJones, AlexandraUberti, FrancescaJones, Arwel W.Ucci, SarassuntaJones, Daniel R.Uddin, Golam MezbahJones, JulieUebanso, TakashiJones, MarjorieUeberschär, OlafJonnalagadda, ShirishaUeno, Hiroshi M.Jonvik, Kristin LundanesUeno, MikinoriJoo, Nam-SeokUeno, ShuichiJorda, Marta AlegretUeno, YujiJose, Pedro A.Ueshima, JunkoJoshi, Shivali S.Uhm, Ju-YeonJoven, JorgeUlatowski, LynnJówko, EwaUlrich, BenjaminJoy, EdwardUlrich, CraigJoy, Jordan M.Ulu, ArzuJu, Sang-YhunUmano, Giuseppina RosariaJu, TingtingUmegaki, HiroyukiJuan, WenyenUmemura, NaokiJuang, Shin-HunUmetani, MichihisaJukić Peladić, NikolinaUmmarino, SimoneJulia, ChantalUmpaichitra, VatcharapanJulien, Jean-PierreUnchwaniwala, NuruddinJulius, UlrichUnger, EricaJulve, JosepUniacke, BlairJun, Hee-SookUnno, KeikoJun, ShinyoungUnno, TatsuyaJun, WoojinUpadhyay, Kiran K.Jung, HeewonUrashima, MitsuyoshiJung, Hyun ChulUrashima, TadasuJung, JooheeUrbanelli, LorenaJung, Kwang-MookUrcuqui-Inchima, SilvioJung, SeungyounUribarri, JaimeJung, YoungSukUrrialde De Andrés, RafaelJunge, CarolineUrschel, KristineJuntti-Berggren, LisaUsai Satta, PaoloJurado Castro, José ManuelUssar, SiegfriedJurado-Fasoli, LucasUusi-Rasi, KirstiJurewitsch, BrianUusitalo, UllaJurjus, Rosalyn A.V Ivanov, AlexanderJuszczak, LesławVacante, MarcoJutand, Marthe-AlineVacca, MicheleJuulskov Holm, LauritsVaccarezza, MauroJyväkorpi, Satu K.Vaccaro, Joan A.Kaaja, RistoVafiadaki, ElizabethKabata, PawelVagianos, KathyKabil, HadiseVahdaninia, MariamKabir, Mohammad FaujulVaidyanathan, VenkateshKabir, MorvaridVaisman, BorisKabisch, StefanVaismoradi, MojtabaKable, Mary E.Vajda, PetrKaburagi, TomokoVakrou, StylianiKačániová, MiroslavaValacchi, GiuseppeKachroo, PriyadarshiniValayannopoulos, VassiliKachuri, LindaValdivielso, Jose ManuelKaczyńska, KatarzynaValencak, TeresaKadela-Tomanek, MonikaValencia, JarisKaden, ReneValensin, DanielaKaeffer, BertrandValente, AnaKaesler, NadineValentine, Christina J.Kagawa, MasaharuValentine, GregoryKagey, Jacob D.Valenzano, AnnaKahlert, RahelValera-Gran, DesiréeKahlon, Talwinder S.Valerio, AlessandraKaido, ToshimiValerio, GiulianaKajarabille, NaroaValero, Javier SanzKaklamanou, DaphneValitutti, FrancescoKalantar-Zadeh, KamyarVallianou, Natalia G.Kalhan, SatishVallone, DanielaKalicki, BolesławValls-Bellés, VictoriaKalin, Marcia F.Vamvakis, AnastasiosKalisz, StanisławVan Ballegooijen, HanneKalliopi, KotsaVan Beusecum, Justin P.Kalman, DouglasVan Bodegraven, Ad A.Kalsbeek, AndriesVan Breda, SimoneKaltsatou, AntoniaVan Buiten, Charlene B.Kam, LynnVan Buul, VincentKamada, KazuhiroVan Calcar, Sandra C.Kamenidou, IreneVan De Heijning, Bert J. M.Kamiloglu, SenemVan Den Abbeele, PieterKamiński, Tomasz W.Van Den Heuvel, Ellen G. H. M.Kamolz, Lars-Petervan der Bend, Daphne L. M.Kampmann, UllaVan Der Kamp, Jan WillemKampouras, AsteriosVan Der Laarse, Willem J.Kamprath, SagarVan Der Merwe, MarieKanakakis, JohnVan Der Wurff, IngeKanakis, GeorgeVan Der Zee, EddyKanauchi, MasaoVan Dijk, Jan-WillemKancherla, VijayaVan Dronkelaar, CarlieneKanczler, Janos M.Van Egmond, LieveKanda, AtsushiVan Elssen, JanineKandikattu, HemanthVan Erp, PietKandylis, PanagiotisVan Gestel, Laurens C.Kaneko, GenVan Greevenbroek, Marleen M. J.Kaneko, KiyokoVan Hemert, SaskiaKanellos, PanagiotisVan Kasteren, PuckKanerva, NooraVan Leeuwen, Sander S.Kang, Hee EunVan Lippevelde, WendyKang, Jae SeungVan Rijn, Bas B.Kang, JeonghyunVan Rijt, LeonieKang, JiheeVan Schaardenburg, DirkjanKang, Min-JungVan Schothorst, Evert M.Kang, WonkuVan Tilburg, Miranda A.L.Kanike, NeelakantaVan Valkengoed, Irene G.M.Kaňková, KateřinaVan Vliet, StephanKantake, MasatoVan Woerden, IreneKantono, KevinVan’t Land, BelindaKao, Li-TingVance, David E.Kao, Shao-HsuanVandenplas, YvanKapahalia, BhupendraVandeputte, DorisKaplan, Joshua M.Vander Wal, Jillon S.Kapravelou, GaryfalliaVander Wyst, KileyKapsokefalou, MaríaVanderhoof, Jon A.Kaput, JimVanderVeen, BrandonKar, SiddharthaVanDusseldorp, TrishaKaraduta, OledVandyousefi, SarvenazKarakiewicz, BeataVaquero-Cristobal, RaquelKaram, SherifVaranoske, AlyssaKaramacoska, DianaVaranoske, Alyssa N.Karaman, SinemVarela, Lourdes M.Karasawa, KenVarese, EricaKarayiannakis, AnastasiosVaresio, CostanzaKarayiannis, DimitriosVarga, BalazsKarbownik, MichałVargas, Ashley J.Karcz, DariuszVargas, Jorge H.Karikas, George-AlbertVargas, PerlaKarimi, KhalilVarillas, DavidKarl, J. PhillipVarriale, SimonaKarlsson, ErikVartak, AbhishekKårlund, AnnaVasconcelos, Manuel PestanaKarmelić, IvanaVasilopoulos, GeorgiosKarolkiewicz, JoannaVassanelli, AuroraKarpe, Avinash V.Vassilakou, ToniaKarppinen, Jari E.Vasta, Florencia C.Karras, Spyridon N.Vasto, SonyaKarunanayake, Chandima P.Vasudevan, AbhinavKarvinen, SiraVasudevan, SreelakshmiKarwowski, BoleslawVatcheva, KristinaKasahara, KazuyukiVäyrynen, Juha P.Kaseva, NinaVazquez-Manrique, RafaelKashiouris, MarkosVeeramani, SureshKašparovský, TomášVeggiotti, PierangeloKasprzak, JaroslawVelagapudi, RavikanthKassan, ModarVelasco, InésKatada, KazuhiroVeleeparambil, ManojKatakam, PrasadVelikova, TsvetelinaKataki, MariaVella, Filomena MonicaKatakura, YoshinoriVemuri, RavichandraKatayama, KazuhiroVendemiale, GianluigiKatayama, ShigeruVenditti, PaolaKato, AkihikoVenegas, EvaKato, HiroyukiVenkatesh, Mohan PammiKato, NorihisaVentimiglia, MarcoKato, TakamitsuVentrella, DomenicoKatsagoni, Christina N.Venturi, ManuelKatsarou, AngelikiVerbeke, KristinKatsarou, Anna-MariaVerd, SergioKattelmann, KendraVerde, ZoraidaKattner, LarsVergalllo, AndreaKatzmarzyk, PeterVergnes, LaurentKauerova, SonaVerhaeghe, JohanKaufman-Shriqui, VeredVerhagen, HansKaur, GunveenVerhulst, StefaanKaushik, Nagendra KumarVerkouter, IngeKaushik, ShellyVerlaan, SjorsKavanagh, Katherine F.Verma, AnilKaviani, MojtabaVerma, Anil K.Kaviani, SepidehVerma, NupurKawabata, KoheiVerma, ShivKawabata, TerueVermeiren, YannickKawakami, RyokoVermiglio, FrancescoKawakita, DaisukeVerna, GiulioKawamoto, NorioVerneau, FabioKawamukai, MakotoVerpeut, JessicaKawamura, AkiraVerri, TizianoKawata, YukichikaVerrotti, AlbertoKay, Colin D.Veselka, BarbaraKaye, GailVeskoukis, Aristidis S.Kazemi, MaryamVest, Katherine E.Kaźmierczak-Siedlecka, KarolinaVetrani, ClaudiaKe, TaoVeysey, MartinKeaver, Laura M.Via, Michael A.Kebbe, MaryamViazis, NikosKędzierska, EwaVicente-Salar, NéstorKeenan, Jacqueline I.Vici, GiorgiaKefleyesus, AmanielVickers, ZataKeiler, Annekathrin MartinaVidgen, HelenKeim, NancyVidot, HelenKelleher, Shannon L.Vidovic, SinisaKeller, AmelieVidyadhara, D. J.Keller, Amélie CléoViegas, CarlaKeller, UlrichVieira, MargaridaKelley, EricVieth, ReinholdKelly, Claire M.Viganò, ElenaKelly, DervlaVignini, AriannaKelly, StephanieViguerie, NathalieKempf, KerstinVila, NataliaKendig, MichaelVilaro, MelissaKennedy, ArionVilaro, Melissa J.Kennedy, LindseyVildmyren, IselinKennel, JulieVilela, SofiaKent, KatherineVillafaina, SantosKeogh, JenniferVillalba, Josè ManuelKeogh, JustinVillani, AnthonyKepka, AlinaVillarini, AnnaKerksick, ChadVillaroya, FrancescKernick, DavidVillaseñor, AlmaKerr, DeborahVincent, AmyKerr, KirkVincent, John BertramKershaw, JonathanVincentini, OlimpiaKerver, JeanVinciguerra, ManlioKervezee, LauraVingolo, Enzo MariaKeshari, SunitaVining, Eileen P.G.Keszler, AgnesVinoy, SophieKesztyüs, DorotheaVinson, JoeKevany, KathleenVionnet, NathalieKhacharem, AïmenVioque, JesúsKhachik, FredVirella, DanielKhajeh, EliasVirsolvy, AnneKhalesi, SamanVirto, MailoKhan, Mohd MohsinViscido, AngeloKhan, NababVisioli, FrancescoKhan, SajidVisser, LydiaKhandelwal, PoojaVissers, MargreetKhandelwal, SoniVisvanathan, RenukaKhanna, SavitaVita, RobertoKharat, SuhasVitacolonna, EsterKhare, SharadVitale, MarilenaKhor, Yet HongVitale, Salvatore GiovanniKhoury, DaliaVitali Čepo, DubravkaKhubchandani, JagdishVitetta, LuisKhullar, SachinVitoria Miñana, IsidroKidoń, MarcinVlassopoulos, AntoniosKieliszek, MarekVlassopoulos, AntonisKiess, WielandVlieg-Boerstra, BerberKiesswetter, EvaVO, EdvardssonKikuchi, ShogoVogt, LiffertKikutani, TakeshiVolaklis, KonstantinosKilari, SreenivasuluVolicer, LadislavKim, Cho-ilVolkert, DorotheeKim, Choon YoungVomhof-DeKrey, EmilieKim, ChunkiVon Döbeln, UlrikaKim, Duk HwanVon Ehrlich, BodoKim, Eun-KyungVon Philipsborn, PeterKim, Gon SupVon Schacky, ClemensKim, Han-NaVon Witzleben, AdrianKim, Hye-KyeongVon Zglinicki, ThomasKim, Hyun SookVonghia, LuisaKim, HyungjinVormann, JürgenKim, HyunjuVoziyan, PaulKim, Il YoungVukasović, TinaKim, JaehanVuksan, VladimirKim, JaekwangVuorio, AlpoKim, Jai-EunWaagbo, RuneKim, JeongseonWada, YasuakiKim, Ji YeonWaddell, JaylynKim, JisuWade, AlexandraKim, Jong-EunWadolowska, LidiaKim, JooyoungWagenmakers, AntonKim, Joo-YoungWägner, Ana M.Kim, JundalWagner, AnikaKim, Jung-HaWagner, MichaelKim, Ju-YoungWagner, RichardKim, Kil-SooWahi, KanuKim, KirangWahlin, StaffanKim, MikyungWaisundara, VidhurangaKim, Min-HyunWakabayashi, IchiroKim, Ok-KyungWakade, ChandramohanKim, Sung-EunWakino, ShuKim, SunheeWaldherr, KarinKim, Tae ImWaldhör, ThomasKim, Won HoWali, JibranKim, Won-YoungWalker, Ashley E.Kim, YanghaWalker, D. CatherineKim, Yeo HyungWalker, Douglas G.Kim, YeonsooWalker, EdwardKim, YongsooWalker, MarvinKim, YoonaWalker, Maura E.Kim, Young-SangWalker, Meegan A.Kimura, YasumiWalker, ValerieKinaciyan, TamarWall, BenKindt, AlidaWallace, AlisonKindy, Mark S.Wallace, MartinaKing, JamesWallace, RuthKiortsis, DimitriosWallace, TaylorKira, GeoffWallenborn, JordynKirby, AnnieWallert, MariaKirkland, Anna E.Walshe, IanKishida, TaroWalter, KathleenKiss, AnnaWalter, OfraKiss, NicoleWalther, WolfgangKiss, RobertWalton, KathrynKitamura, MineakiWalyaro, DJKitaoka, MomokoWalz, KatherinaKitazawa, TakioWalzem, RosemaryKitson, Matthew T.Wanders, DesireeKitta, TakeyaWang, Bo (Australia)Kivelä, LauraWang, Bo (USA)Klaassen, Curtis D.Wang, ChaoKlar, AgnesWang, ChaoChenKleber, Marcus E.Wang, Cheng-HsuKleemann, RobertWang, Chin-KunKlein, Dylan J.Wang, Chung-JenKlein, Gordon L.Wang, DantongKlein-Seetharaman, JudithWang, David Q.Klek, StanislavWang, Ding-HanKlemp, AlexWang, DongqingKleuser, BurkhardWang, DuolaoKlevebro, SusannaWang, Fan-FenKlewicka, ElzbietaWang, FloraKlimina, Ksenia M.Wang, GuanKlímová, BlankaWang, Hui-minKlinger, MarianWang, Jie (Jane)Klonizakis, MarkosWang, JingKlughammer, JohannaWang, KaiKnapik, JosephWang, Kai-LeeKnauf, ClaudeWang, KimberleyKnez, MarijaWang, LiaoKnight, JohnWang, LingKnight, KathyWang, LipingKnipping, KarenWang, Ming-DongKnoop, KathrynWang, Ming-FuKnudsen, Knud Erik BachWang, Mong-HengKo, EunjeongWang, PengchengKo, KwangsukWang, Qian JaniceKob, RobertWang, QingbinKobayashi, SumitakaWang, RuiKobayashi-Hattori, KazuoWang, ShixiongKobus-Cisowska, JoannaWang, Tsung-JenKoch, WojciechWang, WanyiKochmanski, Joseph JohnWang, XianfengKodama, DaisukeWang, XiangbingKoeffel, ReneWang, XiaodongKoehler, EleonoreWang, XiaowanKoelmel, JeremyWang, XiaoxinKoenig, LauraWang, XinKoenigstorfer, JoergWang, XuewenKoga, Jun-ichiroWang, YanwenKogawa, TakahiroWang, YichenKoh, Gar YeeWang, YifeiKoh, Ho-JinWang, Yuh-FengKoh, Jin-HoWang, ZenengKohan, RollandWang, ZheKoike, HarukiWang, ZilongKok, DieuwertjeWangchuk, PhurpaKok, WouterWanich, UrachaKokaze, AkatsukiWanigatunga, Amal AsiriKolasa-Wołosiuk, AgnieszkaWanner, JuergenKolčić, IvanaWanrooij, PaulinaKoli, SwanandWanyonyi, StephenKoliarakis, IoannisWard, Diane McVeyKoliesnik, IevgenWard, LeighKolluru, GopiWard, RobertKolodziejczyk, AleksandraWardowska, AnnaKolodziejski, Pawel AntoniWard-Ritacco, ChristieKolota, AleksandraWarfel, Jaycob D.Kołożyn-Krajewska, DanutaWarkentin, SarahKolwicz Jr., Stephen C.Warner, John O.Komaravolu, RaviWatanabe, MikikoKomarova, SvetlanaWätjen, WimKometani, MitsuhiroWatkins, Adam J.Komnenov, DraganaWatkins, JuliaKomoike, YutaWatso, Joseph C.Kompa, Andrew R.Watson, PoppyKones, RichardWatson, Samuel I.Kong Thoo Lin, PaulWatts, JenniferKong, Byung-WhiWawer, AnnaKong, Kai LingWawrusiewicz-Kurylonek, NataliaKonhilas, JohnWawrzyniak, AgataKönig, DanielWax, BenjaminKonishi, TetsuyaWazed, Md AbdulKonjar, ŠpelaWeatherspoon, LorraineKonopelski, PiotrWeaver, Connie M.Konstantinidis, Theocharis G.Webb, AnnKonstantinidou, ValentiniWebb, BenjaminKonstantinov, Spiro M.Webb, KarenKontek, BogdanWeber, Georg F.Konturek, Peter ChristopherWeber, JanetKoob, GeorgeWeber, LynnKooman, Jeroen P.Weems, SuzyKoon, Hon WaiWeerasekara, IshankaKoopman, ReneWeerasekara, Permani C.Koozehchian, MajidWeger-Lucarelli, JamesKopczynski, MichalWeghuber, JulianKopeć, AnetaWegmüller, RitaKopec, RachelWei, JingKaiKopecky, JanWeickert, Martin O.Kopp Lugli, AndreaWeighardt, HeikeKoppe, Janna G.Weiler, HopeKoppe, LaetitiaWeimann, ArvedKorach-André, MarionWeingärtner, OliverKorczak, ReneeWeir, MichaelKornblith, EricaWeiss, EdwardKorre, MariaWeiss, SiegfriedKortas, JakubWeiss, Thomas S.Korybalska, KatarzynaWeker, HalinaKoshinaka, KeiichiWelham, SimonKosinska-Kaczynska, KatarzynaWeller, AronKosinsky, Robyn LauraWelsh, Jean A.Kosmadopoulos, AnastasiWen, Huei JhenKosteli, Maria ChristinaWen, JianjunKotsa, KalliopiWendell, Stacy GelhausKoturbash, IgorWendy, WismerKot-Wasik, AgataWeng, Ching FengKotzakioulafi, EvangeliaWentz, Laurel M.Kourkouta, LambriniWeres, AnetaKoutelidakis, Antonios E.Werner, TanjaKoutoukidis, DimitriosWernio, EdytaKoutsos, AthanasiosWes, ZimmermannKouzu, HidemichiWeßels, IngaKovarik, MiroslavWesson, DonaldKovatcheva-Datchary, PetiaWest, DanielKoven, Steven G.Westerterp-Plantenga, MargrietKowal, MałgorzataWestmark, CaraKowalczewski, Przemysław ŁukaszWestmark, Cara J.Kowalska, JoannaWetherill, MariannaKowalska, KatarzynaWewalka, MarleneKowalska-Duplaga, KingaWeyermann, MariaKoyama, YuWhatnall, MeganKozak, Andrea T.Wheeler, RichardKozhevnikova, OyunaWheeler, ThomasKozieł, DorotaWhelan, JillKozioł-Kozakowska, AgnieszkaWhipple, Chery A.Kozlowski, Michael R.Whitcomb, Elizabeth A.Kozyrskyj, AnitaWhite, DavidKracht, ChelseaWhite, John H.Krähenbühl, StephanWhite, Robin E.Krajka-Kuźniak, ViolettaWhitfield, KylyKramer, EllenWhiting, SusanKramer, Eydie N.Whittington, JenniferKranz, SibylleWhybrow, StephenKranzler, MarkusWiatrak, BenitaKrause, Matthew P.Wiciński, MichałKreider, RichardWiczkowski, WieslawKrejpcio, ZbigniewWie, Myung-BokKrela-Kaźmierczak, IwonaWięcek, MagdalenaKrishna, GokulWiechmann, AllanKrishnamoorthy, LakshmipriyaWięckowska, BarbaraKrishnaswamy, KirubaWiedemann, AshleyKristo, AleksandraWierdak, MateuszKristof, Arnold S.Wierzbicka, ElżbietaKroeger, Cynthia M.Wierzejska, ReginaKroeze, WillemiekeWiggs, MichaelKról, EwelinaWightman, Emma L.Królak-Olejnik, BarbaraWilkinson, LauraKroon, PaulWillems, Mark Elisabeth TheodorusKrot, KatarzynaWillette, Auriel A.Krüger, Renata L.Williams, ElizabethKrukowski, RebeccaWilliams, JamesKrupa-Kozak, UrszulaWilliams, Jessica A.Krusinska, BeataWilliams, SusanKrzeptowski, WojciechWillis, DonKrzesiński, PawełWilson, AnnabelleKrzysztof Nowak, JanWilson, CallumKrzysztofik, MichałWilson, JacobKrzyżanowski, ArkadiuszWilson, Jeffrey M.Książek, AnnaWilson, R. DouglasKsiazyk, JanuszWilson, Robin A.Ku, SeockmoWilson, TedKuang, JujiaoWilson, Thomas A.Kubal-Czerwińska, MagdalenaWilson-Barnes, Saskia L.Kubant, RuslanWinchester, LauraKubena, KarenWinckel, Myriam VanKubina, RobertWindler, EberhardKubis, Hans-PeterWingren, Carl JohanKubo, YoshinaoWinham, Donna M.Kubota, MasaruWinkelmann, ZacharyKubota, MegumiWinkels, RenateKubota, ToshiakiWinkler, Marion F.Kubrak, CatherineWinn, NathanKüchle, ClaudiusWinneke, GerhardKuczmarski, MarieWinsz-Szczotka, KatarzynaKudo, TakashiWinters, BryonyKufer, T. A.Winters, StephenKuhla, AngelaWinters-van Eekelen, EstherKuk, Jennifer L.Winuthayanon, WipaweeKulaga, ZbigniewWirnitzer, KatharinaKulczyński, BartoszWirth, MichaelKulkarni, ShardulWirth, RainerKulma, AnnaWisnieski, LaurenKumar, AmitWiszumirska, KarolinaKumar, AnandWitard, OliverKumar, AvinashWithrow, NicoleKumar, PradeepWitkowska, Anna MariaKumar, PraveenWitkowska-Zimny, MalgorzataKumar, RajWitkowski, StanisławKumar, VijayWitrick, KatherineKumrungsee, ThanutchapornWitt-Enderby, PaulaKunces, Laura J.Wittrant, YohannKung, Woon-ManWłodarczyk, MaciejKunugi, HiroshiWłodarczyk, MartaKuo, Chia-FengWłodarek, DariuszKuo, TonyWlodek, MaryKuphal, SilkeWnuk, Susan MargaretKuppusamy, ManiselvanWoessner, Mary N.Kurada, LalithaWoith, EricKurath-Koller, StefanWojciechowska-Solis, JuliaKurian, SunilWojcik, CezaryKurien, Biji TheyilamannilWojcik, Janet R.Kurihara, NobutakaWójcik, MałgorzataKurilshikov, AlexanderWojnar, WeronikaKurowski, MarcinWojszel, Zyta BeataKurppa, KalleWojtunik-Kulesza, Karolina A.Kurrpa, KalleWoldeamanuel, Yohannes WoubishetKurth, SalomeWolever, ThomasKuryłowicz, AlinaWomack, Christopher J.Kurz, KatharinaWong, AlexeiKus, PiotrWong, AnnetteKushida, OsamuWong, JuliaKushkevych, IvanWong, Te-ChihKussmann, MartinWong, YUjunKuula, LiisaWood, Charles E.Kuwana, TomomiWoodfield, LorayneKwak, Hyun JeongWooding, StephenKwan, Sze Ting (Cecilia)Woodman, RichardKwon, HyokjoonWoodruff, BradleyKwon, JaewooWoods, JulieKwon, Ki-SunWoodside, JayneKwon, OranWoodward, BillKwon, Tae-HwanWoodward, JeremyKwon, Yu-jinWootan, MargoKwun, In-SookWooten, Joshua S.Kypreos, KyriakosWoźniak, LucynaKyrgios, IoannisWoźniak, ŁukaszKyriacou, AdamantiniWright, Charlotte M.Kyrou, IoannisWright, ChristianL. Wardle, SophieWright, ChristineLa Frano, MichaelWright, HattieLa Frano, Michael R.Wright, Kenneth P.La Manna, GaetanoWright, OliviaLa Mendola, DiegoWright, PatrickLa Spina, MartinaWrześniok, DorotaLa Vecchia, CarloWu, CenLa Vieille, SébastienWu, Chieh-ChenLaaksonen, VanessaWu, Chun-ChiLaatikainen, ReijoWu, GraceLabeit, SiegfriedWu, Hung-TsungLacatusu, Cristina MihaelaWu, I-ChenLăcătușu, Cristina-MihaelaWu, Jia PingLach, GilliardWu, JunxiLacham-Kaplan, OrlyWu, Li-WeiLachance, Laura R.Wu, Meng-YuLachenmeier, DirkWu, RaymondLachlan, MitchellWu, RenyiLachmann, NicoWu, Richard YouLachowicz, SabinaWu, Tzu-HuaLacombe, ScottWu, Wei-LiLaffin, MichaelWu, Wen-TzuLaganà, Antonio SimoneWu, XianLagarde, MichelWu, YouŁagowska, KarolinaWu, ZhihaoLaguna, AriadnaWulff, Anders BergLaguna, LauraWunderlich, Shahla M.Lahl, KatharinaWyganowska-Świątkowska, MarzenaLahooti, HooshangWyse, RebeccaLahtinen, MariaXepapadaki, ParaskeviLai, AntoniaXia, GuobinLai, Chien-HanXia, YinglinLai, Ching-ShuXiao, JianqiuLai, I-JuXiao, QianLai, SilviaXiao, XiaLai, YandongXiaoli, AlusLaimer, JohannesXie, BingxianLaird, EamonXie, MouzheLajtai, KrisztinaXie, WankunLalia, Antigoni LaliaXu, ChuanmingLama, AdrianoXu, GegeLamagna, BarbaraXu, JingLambert, ElisabethXu, PengfeiLambert, JoshuaXu, ZhihongLambert, KarenXue, HongLambert, KellyXue, XiangLambertini, ElisabettaYadav, DhananjayLamers, YvonneYamada, HiroshiLamichhane, SantoshYamada, SatoruLamminpää, ReetaYamaki, KouyaLamore, KristopherYamamoto, KouichiLanaspa, MiguelYamamoto, SuguruLand, Mary-AnneYamamoto, ToshiharuLandberg, RikardYamanaka, GakuLandete, José MariaYamaoka, YusukeLandi, MassimoYamasaki, MasahiroLandry, GregYamashiro, YuichiroLandry, Matthew J.Yamashita, ShinjiLands, Larry C.Yamauchi, HidekoLane, GinnyYamazaki, TomomiLang, ChristinYan, Alice FangLang, SonjaYan, Liang-junLange, EwaYan, MaryLangeard, AntoineYan, ShengminLanigan, Jane D.Yan, TingtingLapaquette, PierreYan, XuLaparra, Jose MoisésYan, Xu SeanLaPointe, GisèleYanagisawa, DaijiroLappa, IliadaYanaka, NoriyukiLarsen Tromso, TerjeYanase, SuminoLarsen, JunillaYang, Ai-LunLarussa, TizianaYang, Angela Wei HongLasekan, John B.Yang, BoLaselva, OnofrioYang, Chang-HaoLasker, JordanYang, Cheng-YaoLasonder, EdwinYang, Chung ShuLasrado, Joanne A.Yang, Feili LoLassemillante, Annie-ClaudeYang, GabsikLatino, Maria ElenaYang, GuangLattanzi, SimonaYang, Hsin-YiLatunde-Dada, Gladys OluyemisiYang, Hui-TingLau, ChunghoYang, JiminLau, VivianYang, KemingLauren, AuYang, Kuen-ChehLauria, FabioYang, KunLaurikka, PilviYang, MikeLauritzen, LotteYang, QiyuanLaursen, Martin FrederikYang, Rong-SenLavelle, FionaYang, Seung HwanLavelli, VeraYang, ShiLavender, AndrewYang, Shwu-HueyLaviano, AlessandroYang, Soo JinLavie, Carl J.Yang, Suh-ChingLavoie, PascalYang, Wai YewLaVoy, EmilyYang, Wei-hsiungLavriša, ŽivaYang, YongkangLawen, AlfonsYang, YueLawson, CharlotteYang, ZhihongLawson, KimYannicelli, StevenLawson, VictoriaYano, ShojiLayman, DonaldYano, ShozoLayman, Donald K.Yao, JiayiLazarevic, VladimirYasar, ZerbuLe Dréan, GwenolaYasuda, JunLe Foll, ChristelleYasuko, ManabeLe Guillou, DouniaYavropoulou, Maria P.Le Joncour, VadimYaxley, AlisonLe Magueresse-Battistoni, BrigitteYayan, JosefLe Port, AgnesYe, KaixiongLe Thanh-Blicharz, JoannaYe, MingyuLea, Michael A.Yee, Callista StephanieLeach, Steven T.Yee, KimboLebeck, JanneYeger, HermanLebenthal, YaelYeh, Ching-HuaLeber, BettinaYeh, Kun-yunLecerf, Jean-michelYeh, Kuo-WeiLechner, John F.Yen, Cheng-ChiehLechuga-Sancho, Alfonso M.Yen, Cheng-fangLeclerc, EstelleYenerall, JackieLedder, OrenYeo, SujungLedeganck, Kristien J.Yeretzian, ChahanLederer, Ann-KathrinYeruva, LaxmiLederer, Eleanor D.Yi, Dae YongLee, Anne RolandYiannakopoulou, EugeniaLee, Chien-HungYilmaz, BahtiyarLee, Chong JaeYim, Jung-EunLee, Choong-GuYing-ting, CaoLee, Daniel T.Yip, Bon HamLee, DonghunYokoi, KatsuhikoLee, Eugene S.Yokomichi, HiroshiLee, EuniYokoyama, YokoLee, GihyunYoo, Hah YoungLee, GunaYoo, Hye JinLee, Hong-JinYoo, Ji YounLee, Hye-JaYoo, Seung-HeeLee, JaecheolYoo, So YoungLee, Jae-GilYoon, CynthiaLee, JaeminYoon, UzungLee, JenniferYoshida, MitsuyoshiLee, JoohyongYoshida, NaofumiLee, Jun SikYoshida, TakahikoLee, Jung EunYoshihara, ToshinoriLee, KeehoonYoshikawa, HiroakiLee, Kun-HuaYoshikawa, ToruLee, Kwang-GeunYoshikazu, NakamuraLee, Mei-hwaYoshimura, YoshihiroLee, Mei-YuehYoshioka, YasukiyoLee, Myoung-SookYoshizawa, KazukoLee, Sang-ahYou, Hyun JuLee, Seung-YeonYoun, Hee-ShangLee, Soo-younYoung, Howard A.Lee, Sung KiYoung, Lauren M.Lee, Sung-HyenYounge, Noelle ElizabethLee, SunjaeYoung-Hwan, JoLee, Teng-YuYu, HongLee, VincentYu, JiujiuLee, Yen-HanYu, XiaohuaLee, Yoon KwangYu, YanboLee, YoonJungYu, YanpingLee, YunhwanYu, ZhanyangLee, YunkyoungYu, ZhijieLeemaqz, ShalemYuan, Kuo-ChingLees, MatthewYuan, LiyunLee-Sarwar, Kathleen AnneYuan, QiLeeth, CarolineYuann, Jeu-Ming P.Lefebvre, David E.Yui, KunioLefere, SanderYumoto, HiromichiLegetic, BrankaYun, Jong WonLegg, EricYurko-Mauro, KarinLehmann, UndineYzydorczyk, CatherineLehtovirta, MikkoZabet Moghaddam, MasoudLei, BenfangZabetakis, Ioannis A.Lei, WeiZabłocka-Słowińska, KatarzynaLeibovitz, EyalZaborina, OlgaLeibowitz, AvshalomZacek, PetrLeigh, JamesZachariassen, GitteLeikin-Frenkel, Alicia I.Zafrilla, PilarLeimanis, MaraZagarins, SofijaLeiphrakpam, PremilaZagórska, AgnieszkaLelli, DianaZaguła, GrzegorzLemacks, JenniferZaharia, SoniaLemay, DanielleZahradka, PeterLemieux, HélèneZaibi, Mohamed SghaierLemon, PeterZaiss, MarioLemos, Bruno S.Zajac, Ian TaylorLemos, Bruno SilvaZakłos-Szyda, MalgorzataLems, WillemZakrocka, IzabelaLenaerts, KaatjeZalewska, AnnaLennon, ShannonZalewska, MagdalenaLenzi, JacopoZalewski, Bartłomiej M.Leo, Alfredo DiZama, DanieleLeo, Chen HueiZaman, SarahLeong, ClaudiaZambrano, Rodrigo ElíasLeonn Guerrero, RachaelZammit, Stefania ChetcutiLeopold, Jane A.Zamora-Ros, RaulLeow, MelvinZand, NazaninLerchbaum, ElisabethZanella, IsabellaLesgards, Jean-FrançoisZang, LiqingLeshem, Micah B.Zanini, BarbaraLesicka, MonikaZanon, Renata GracieleLeszczak, JustynaZanon-Moreno, Vicente CalixtoLeszczyńska, TeresaZanotti, IlariaLevenson, CathyZapata, RizaldyLeventakou, VasilikiZara, VincenzoLevin, SusanZaragoza Martí, AnaLevine, ArieZárate, RafaelLevolger, StefZariwala, Mohammed GulrezLevy, EmileZarnowski, TomaszLevy, LouisZarobkiewicz, MichałLewandowicz, JacekZarrazquin, IdoiaLewandowska, MałgorzataZart, SebastianLewicka, AnetaZarzycki, PiotrLewicki, SławomirZastre, JasonLewis, Erin DianeZatterale, FedericaLewis, JoZatwarnicka-Madura, BeataLewis, StephenZaura, EgijaLewthwaite, HayleyZavattari, PatriziaLeyton Roman, MartaZawadzka, MalgorzataLeyuan, LiŻbikowska, AnnaLeyva, Misti J.Zdybel, EwaLi, BinxingZeibich, LydiaLi, Chia-JungZekovic, MilicaLi, ChunyingZeman, DanielLi, Dian-JengZeng, BijunLi, HuaZentek, JuergenLi, HuiZeppieri, MarcoLi, HuinanZerón-Rugerio, María FernandaLi, JiaZeuner, BirgitteLi, JieZevallos, Victor F.Li, JoanZgaga, LinaLi, KeshuaiZhang, BoLi, MiaomiaoZhang, DeliangLi, MinZhang, DeqiangLi, QiongyuZhang, DongyuLi, Ren-keZhang, GuanshiLi, ShizhaoZhang, GuodongLi, ShuoZhang, HuiliangLi, ShushunZhang, JiLi, SunanZhang, JianminLi, WeihanZhang, LingboLi, Wen-WuZhang, LuLi, XiangZhang, QianLi, XinZhang, ShuLi, XinyiZhang, TuoLi, YanqunZhang, XiaotaoLi, YaoZhang, XinxingLi, YihangZhang, XiujuanLi, ZhijunZhang, YixuanLiakopoulos, VassiliosZhang, ZhiguoLian, Ian Y.Z.Zhang, ZhiyingLiang, JunZhao, BaoyinLiang, ZhibinZhao, NingningLiani, RossellaZhao, WeiLiao, Kai-WeiZhao, XiaoaiLiao, PanZhao, YuLiao, Yi-HungZhao, YutongLiao, Yi-JenZheng, HaotianLiao, Yung-FengZheng, JoleneLiao, ZehuanZhong, WeiLiberato, Selma C.Zhou, HongyiLicciardi, PaulZhou, PhoebeLichtveld, KimZhou, QingyuLicini, CaterinaZhou, YanjiaoLieb, WolfgangZhou, ZhenqiLiébana-Presa, CristinaZhu, BingLiebman, EliZhu, ChenghaoLieder, BarbaraZhu, DongshanLieffers, JessicaZhu, LinLiévin-Le Moal, VanessaZhu, QifanLightfoot, Adam P.Zhu, XuejunLiguori, GiorgiaZiarno, MałgorzataLiktor-Busa, ErikaZiauddeen, NidaLiljeberg, EvelinaZięba-Przybylska, DorotaLim, Jeong-HoonZiegenfuss, TimLim, KarenZiegler, AmandaLim, KyuZiegler, Ekhard E.Lim, Stephen E. R.Ziegler, JaneLima, ElisabeteZielińska, DorotaLimdi, JimmyZielinska, MagdalenaLin, Chieh-HsinZielińska, Monika A.Lin, Chih-LiZieliński, DamianLin, Chih-MingZielinski, TomaszLin, Chin-LonZinn, CarynLin, Chung-Tung J.Zitt, EmanuelLin, Chung-YingZitterl-Eglseer, KarinLin, Chun-PingZłotkowska, DagmaraLin, DingboZmijewski, MichalLin, Hsin-TangZnamirowska, AgataLin, Hui-HsuanZoll, JoffreyLin, Jer-AnZorbas, ChristinaLin, JessicaZordoky, BeshayLin, Jian-MingZorrilla, EricLin, Jin-SengZou, PingLin, Li-YinZuercher, JenniferLin, Ming-HongZuhl, MicahLin, Muh-ShiZukow, WaleryLin, Pao-HwaZupunski, LjubicaLin, Pi-I. D.Zwetsloot, KevinLin, Ro-TingZych, MariaLin, Shih-Yi


